# Influence of Environmental Factors on the Production of Penitrems A–F by *Penicillium crustosum*

**DOI:** 10.3390/toxins9070210

**Published:** 2017-07-01

**Authors:** Svetlana A. Kalinina, Annika Jagels, Benedikt Cramer, Rolf Geisen, Hans-Ulrich Humpf

**Affiliations:** 1 Institute of Food Chemistry, Westfälische Wilhelms-Universität Münster, Corrensstraße 45, 48149 Münster, Germany; s_kali03@wwu.de (S.A.K.); a_jage01@uni-muenster.de (A.J.); cramerb@uni-muenster.de (B.C.); 2 NRW Graduate School of Chemistry, Wilhelm-Klemm-Str. 10, 48149 Münster, Germany; 3 Department of Safety and Quality of Fruits and Vegetables, Max Rubner-Institute, Haid-und-Neu-Str. 9, 76121 Karlsruhe, Germany; rolf.geisen@mri.bund.de

**Keywords:** *Penicillium crustosum*, penitrem, flash chromatography, stress factor, cheese model medium, micro-scale extraction, liquid chromatography

## Abstract

Filamentous fungi produce a multitude of secondary metabolites, some of them known as mycotoxins, which are toxic to vertebrates and other animal groups in low concentrations. Among them, penitrems, which belong to the group of indole-diterpene mycotoxins, are synthesized by *Penicillium* and *Aspergillus* genera and exhibit potent tremorgenic effects. This is the first complex study of the penitrems A–F production under the influence of different abiotic factors, e.g., media, incubation time, temperature, pH, light, water activity, and carbon and nitrogen source as well as oxidative and salt stress. For this purpose, penitrems A–F were isolated from *Penicillium crustosum* cultures and used as analytical standards. Among the carbon sources, glucose supplemented to the media at the concentration of 50 g/L, showed the strongest inducing effect on the biosynthesis of penitrems. Among nitrogen sources, glutamate was found to be the most favorable supplement, significantly increasing production of these secondary metabolites. CuSO_4_-promoted oxidative stress was also shown to remarkably stimulate biosynthesis of all penitrems. In contrast, the salt stress, caused by the elevated concentrations of NaCl, showed an inhibitory effect on the penitrem biosynthesis. Finally, cheese model medium elicited exceptionally high production of all members of the penitrems family. Obtained results give insides into the biosynthesis of toxicologically relevant penitrems A–F under different environmental factors and can be utilized to prevent food contamination.

## 1. Introduction

Filamentous fungi produce a multitude of low-molecular-weight compounds known as mycotoxins, which are toxic to vertebrates and other animal groups in low concentrations, causing acute as well as chronic diseases. Among them, indole-diterpenes represent a structurally diverse group of mycotoxins exhibiting potent tremorgenic effects [1,2,3,4]. Whereas a cyclic diterpene scaffold adjacent to an indole heterocycle characterizes the general structure of this group of toxins, their structural variety is achieved by prenylations, different patterns of ring substitutions and the stereochemistry of these substituents [5,6,7,8,9]. 

**Figure 1:**
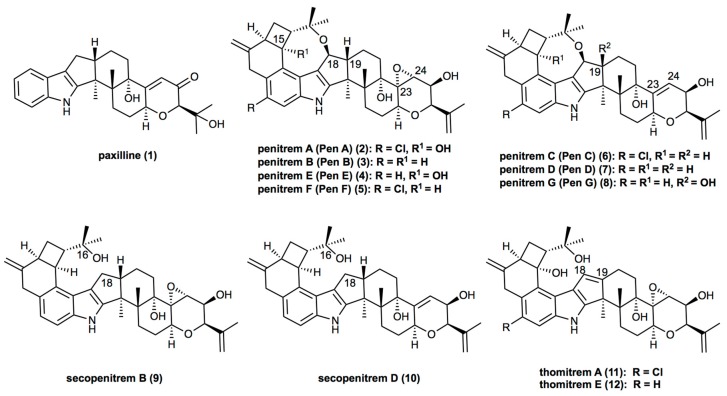
Structures of penitrems and their analogs.

Being paxilline-like (**1**) indole-diterpenes, penitrems (**2**–**8**) exhibit structural diversity, which is characterized by various functional groups attached to the common molecular skeleton. Thus, three members of the family, penitrems A, C, and F, are chlorinated (Figure 1). Moreover, penitrems A and E possess a hydroxy group in position C-15 of the cyclobutyl unit. Whereas penitrems C and D exhibit a double bond in their tetrahydropyran ring (between C-23 and C-24), other penitrems possess an epoxide group at this position (Figure 1). Along with the main members of the family (penitrems A–F), other representatives, e.g., penitrem G, possess a hydroxy group at C-19 [10]. Structural analogs of penitrems, lacking the C-16–C-18 ether linkage are known as secopenitrem B (**9**) [11] and D (**10**) [12], as well as thomitrem A (**11**) and E (**12**) [13], in the case of an additional unsaturated bond between C-18 and C-19 [5,14,15]. 

Unique structural features of penitrems define their exceptional biological activity profile as well as their toxic effects. Thus, for instance, penitrems A–F and their brominated analogs demonstrate anti-proliferative, anti-migratory, and anti-invasive effects in mammary cancer cells [16,17]. Furthermore, penitrems A–D and F show convulsive and insecticidal activities against two studied insect species [10]. Noteworthy, the chlorinated penitrems were found to show the highest insecticidal activity. Several facts support the assumptions that the insects’ mortality is related to the chlorine atom, whereas the epoxy group is affecting the delayed toxicity [10].

Being the most studied compound, penitrem A is known to target the central nervous system via several pathways. It was shown that the toxin increases the spontaneous release of endogenous glutamate, γ-aminobutyric acid (GABA), and aspartate from cerebrocortical synaptosomes, but not from spinal/medullary cord [18]. Moreover, penitrem A inhibits the high-conductance Ca^2+^-activated K^+^-channels (BK) in low concentrations (10 mM) [19]. In addition, penitrem A is able to cross the hematoencephalic barrier readily accessing the CNS (central nervous system) [20]. Finally, in rats, penitrem A has been shown to be the reason for extensive dose-dependent loss of Purkinje cells and also to affect other cerebellar neurons, e.g., granule, basket and stellate cells [21]. 

Along with the experimentally obtained biological activity data, several cases of poisoning have been reported in recent years, evidencing the high toxicity profile of penitrems. Thus, the first case of the occurrence of penitrem A in food was observed in 1979, when moldy cream cheese was suspected to be involved in the intoxication of dogs [22]. Richard and Arp reported a series of intoxication symptoms including generalized convulsions, ataxia, urination, defecation, polypnea, hyperthermia and mydriasis in a dog after consumption of moldy walnuts. In this case, *P. crustosum* was identified as the producer of penitrem A [23]. Several similar cases have been reported in the United States, Australia, South Africa and Norway [24,25,26]. Moreover, penitrem A was suggested to be a reason of the incoordination syndrome, known as ryegrass staggers in New Zealand [27].

Penitrem A intoxication has been well documented in animals, and in 2005, it became clear that the ingestion of food contaminated with penitrem A leads to tremor in humans. Along with contaminated food, toxins of this type could enter the human body via inhalation of spores and hyphal fragments, thereby reaching the bloodstream to be distributed systematically. Furthermore, this route provides direct access for the toxins to the CNS axonal transport along the olfactory nerves, the first cranial entry without protection by the BBB [28].

Fungi that are known to produce penitrems are limited to a relatively small number of species and include *Penicillium* and *Aspergillus* genera [8]. Among them, *P. crustosum* is known to be the main producer of penitrem A, from which it was first isolated in 1968 with the name tremortin A as the most significant metabolite [29]. A number of preparative isolation approaches for penitrems have been reported in the last decades. In most cases, *P. crustosum* is cultivated on Czapek–Dox broth supplemented with yeast extract followed by extraction of the mycelium with organic solvents. Subsequent separation and purification of penitrems is achieved via column chromatography and preparative HPLC [5,10,15,30,31].

Adaptation to different stress factors is needed for parasitic fungi to survive the on-going defense attacks by the host organisms, like plants or humans, and often results in the production of specific secondary metabolites. Furthermore external parameters like substrate composition, temperature, pH- or a_w_ values can have a strong influence on mycotoxin biosynthesis [32]. Thus, to produce secondary metabolites (mycotoxins) in high yields under artificial conditions, the induction of stress and stress adaptation are also relevant factors [33]. However, only a few studies on the influence of different environmental factors and media supplements on the production of penitrems have been reported. For instance, Wagener et al. showed an increase of penitrem A production in cultures of *P. crustosum* grown on YES-medium when the pH is elevated to pH ≈ 9. Moreover, the optimum temperature for growth and toxin production was reported to be 20 °C after 28 days of cultivation. Relative humidity of 99% was also found to be optimal for penitrem A biosynthesis [34]. Notably, various substrates, e.g., corn, wheat, oats, barley, pecans, sausages, dried beef, cheese, peanuts and cottonseed, were reported to be supportive for the production of penitrem A by several fungal species [34,35,36,37]. An addition of yeast extract to the medium was also reported to be crucial for penitrem A production by *P. crustosum* when synthetic growth media were applied [36]. 

For the production of penitrem B by *P. aurantiogriseum*, different synthetic media, supplemented with various nitrogen and carbon sources, have been examined. In this study, D-xylose was found to induce the maximum penitrem B production, whereas glycerol, citric acid and succinic acid had negative effects [38]. The same authors reported an increased penitrem B production by *P. aurantiogriseum*, grown on yeast extract medium, supplemented with sucrose and KNO_3_ [39]. On the other hand, the addition of CaCl_2_ to the medium was found to induce fungal sporulation and the production of penitrems A, C, and E in significant yields in cultures of *P. nigricans*, which are usually known for the production of griseofulvin [40].

The high toxicity profile of penitrems and their occurrence in commercial foodstuff indicates an importance of penitrem production studies for two reasons. On the one hand, evaluation of environmental factors, influencing penitrem formation, is beneficial for predicting food contamination. On the other hand, the optimal conditions for penitrem production are important for their isolation in high yields to be used as analytical standards. However, to date, no complex study of various stress factors, affecting the fungal growth and production of the whole members of the penitrem family, is available in the literature.

The present study investigates the optimal growth conditions for the quantitative production of penitrems A–F by combination of different abiotic factors such as media, incubation time, temperature, pH, light, water activity, carbon and nitrogen source as well as oxidative and salt stress. Moreover, in this study, an additional emphasis was placed on the efficient isolation of penitrems A–F as analytical standards.

## 2. Results

### 2.1. Penitrem A Production Depending on Media, Time, Temperature, pH, and Light

**Figure 2:**
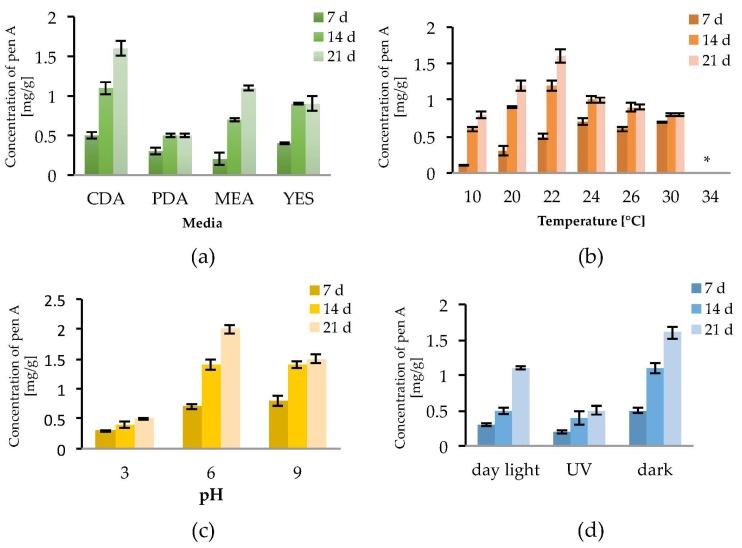
Effect of: media (**a**); temperature (**b**); pH (**c**); and light (**d**) on Pen A (Penitrem A) production by *P. crustosum* after 7, 14 and 21 days of incubation in mg/g dry mass, * no penitrems were detected. Results are mean of five replicates ± standard deviation and given in mg mycotoxin related to g dry mass (mg/g).

Mycotoxin production depends on the fungal strain, the substrate and the environmental conditions. To determine the optimal growth conditions for the production of penitrems A–F, several abiotic factors were screened using *P. crustosum*, which is considered the main producer of penitrems [5,30]. Initially, for monitoring positive or negative effects of abiotic factors on the penitrem production, only the penitrem A concentration, expressed as mg per g of dry biomass, was determined by means of an HPLC-DAD method (Figure 2). This screening was focused on penitrem A as it is the main member of the family, besides penitrem E, which is the final product of the biosynthetic pathway of the penitrems [41]. 

To observe the effect of the incubation time on the production of penitrem A (Pen A), the culture was analyzed after 7, 14 and 21 days of incubation (Figure 2a–d). Irrespective to media composition, temperature, pH, and light exposure, a constant increase of the Pen A concentration was observed between Day 7 and Day 14 of incubation. The concentration continued to grow up to Day 21, however this increase was not significant in all performed experiments (Figure 2a–d). Additionally, it was found that detectable amounts of Pen A could be observed after three days of growth, whereas incubation for more than 21 days reveals only insignificant increment of the toxin concentration (data not shown).

As substrate composition might be critical for the penitrem production, four different media were tested. Among them, Czapek–Dox agar (CDA) was found to be the most favorable for Pen A synthesis as, irrespective to cultivation time, *P. crustosum* produced higher concentrations of Pen A growing on CDA compared to growing on PDA, MEA, or YES medium (Figure 2a and Figure S1a). In order to evaluate the influence of temperature on the penitrem production, the biosynthesis of Pen A was monitored at seven different temperatures in a range between 10 °C and 34 °C (Figure 2b and Figure S1b). It was found that at temperatures between 10 °C and 30 °C *P. crustosum* actively produces Pen A, whereas no production was observed at 34 °C. As in previous studies penitrems were isolated, when *P. crustosum* was grown at 25 °C [42], the temperature interval between 20 °C and 26 °C was studied in details. In this temperature range, an ambiguous effect was observed: after seven days of incubation at 24–30 °C the concentration of Pen A was higher than at other temperatures, while after 14 and 21 days of cultivation, growth at 22 °C yielded the highest levels of Pen A.

To study the influence of pH of media on the penitrem production, fungi were grown under the acidic (pH = 3), neutral (pH = 6), or basic (pH = 9) conditions over a period of 21 days. Although no significant alteration of fungal growth was observed within the range of tested pH, penitrem production changed upon transition from the neutral pH value (Figure 2c and Figure S1c). Thus, maximum accumulation of Pen A was registered at pH 6 over 21 day of incubation. When comparing with neutral pH, acidic pH was found to be detrimental for the Pen A biosynthesis in every tested time period, whereas shift to basic pH value resulted in little to no alteration of Pen A production after 7 and 14 days of incubation. However, after three weeks of growth at basic pH, *P. crustosum* was found to produce around 25% less of Pen A, compared to the toxin production at neutral conditions (Figure 2c).

In addition, exposure to light was found to influence the growth of *P. crustosum* as well as the biosynthesis of Pen A. In general, when growing in dark, the fungi demonstrated the fastest biomass increase and highest Pen A levels. Whereas exposure of *P. crustosum* to daylight only slightly decreased Pen A production, UV irradiation significantly reduced the biosynthesis of this toxin (Figure 2d and Figure S1d). 

**Figure 3:**
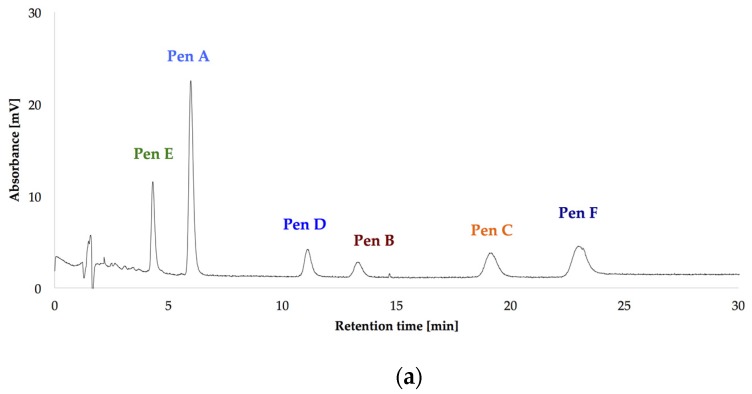

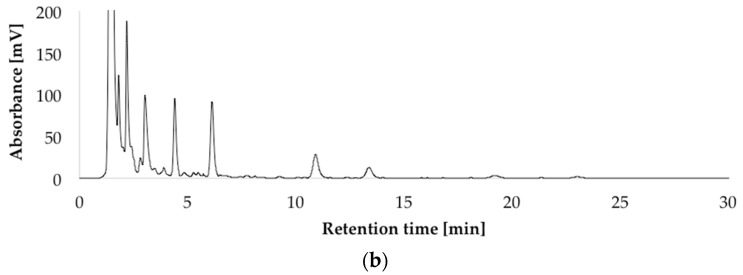
HPLC-UV-DAD (high performance liquid chromatography with ultraviolet diode array detection) chromatograms at 236 nm of the isolated penitrems A–F (10 μg/mL) (**a**); and of *P. crustosum* crude extract cultured on modified CDA (Czapek–Dox agar) supplemented with 15 g/L of glycerol (**b**).

After optimizing the growth conditions for Pen A production (incubation during three weeks in dark on CDA medium at 22 °C, “standard conditions”), the biosynthesis of all members of the penitrem family was studied under these conditions (Figure 3a). For this purpose, penitrems A–F were first isolated from *P. crustosum* cultures growing under the optimized developed method. Briefly, *P. crustosum* was cultivated on CDA medium during three weeks, followed by extraction with 90% acetonitrile. After a concentration step, the crude extract was subjected to flash chromatography with subsequent final separation of the penitrems by preparative HPLC-UV. The structures of the isolated penitrems were proven by spectroscopic methods and were in agreement with previously reported data [42,43] (see Tables S1 and S2 in the Supplementary Materials). Penitrems A–F with a purity of ≥96% were obtained and applied as external standards for the subsequent quantitative analysis (Figure 3a,b).

**Figure 4:**
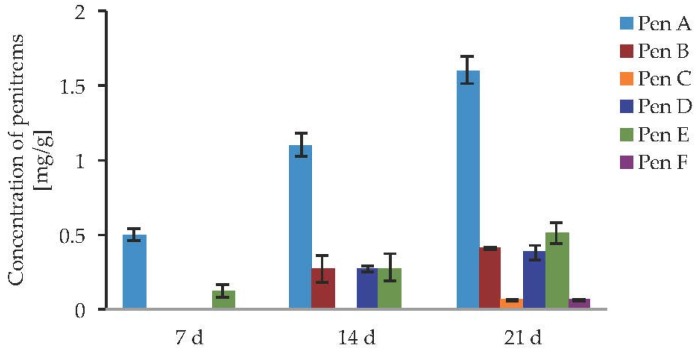
Production of penitrems A–F (mg/g dry mass) after 7, 14 and 21 days of incubation by *P. crustosum* cultured on CDA in the dark at 22 °C (standard conditions). Results are mean of five replicates ± standard deviation and given in mg mycotoxin related to g dry mass (mg/g).

After the first week of *P. crustosum* cultivation under the standard conditions, only penitrems A and E could be detected (Figure 4, Table S3) while at the end of the second week two more members of the penitrem family, penitrems B and D, were formed in concentrations equal to Pen E. While Pen A at that point reached a concentration of approximately 1.2 mg/g, the others three showed equal concentration of about 0.27 mg/g. All penitrems were observed only after 21 days of incubation. Pen A as the main compound was detected in a high yield of 1.6 mg/g, followed by Pen E, which production was about four times lower (0.51 mg/g) and comparable to concentrations of Pen B and Pen D. Being produced only after the third week of incubation, Pen C and Pen F were only detected in concentrations as low as 0.06 mg/g each.

### 2.2. Impact of the Carbon Source on the Production of Penitrems A–F

Carbon source might be a limiting factor of fungal growth as well as of toxin production. To study the impact of this factor in detail, *P. crustosum* was cultivated in Petri dishes with media supplemented with different carbon sources, and concentrations of penitrems A–F were measured after 7, 14 and 21 days (Table S4, Figure S2). To minimize the impact of potential additional nutrient sources, yeast extract was excluded from the media. The results after 21 days of incubation are summarized in Table 1. 

Media, which were modified with starch, sorbitol and cellulose, elicited the lowest penitrems production level after 21 days of cultivation. Moreover, mentioned supplements were not able to lead to the production of penitrems C and F. In contrast, *P. crustosum* grown on media supplemented with more bioavailable mono- and disaccharides, produced all penitrems in higher concentrations. Particularly, glucose and rhamnose were found to be the most favorable supplements for penitrem production, for instance leading to 1.13 mg/g and 0.95 mg/g of Pen A concentrations after 21 days of incubation, respectively (Table 1). It was also observed that *P. crustosum*, cultivated on glucose-supplemented medium, produces around four times higher concentrations of Pen A than of Pen D and Pen E, whereas concentration of Pen B is twice less than the latter two. Under the same conditions, Pen C and Pen F were produced in even lower and hardly detectable concentrations. Interestingly, the ratio between all members of the penitrem family, irrespective to the carbon source, was constant and similar to those observed under the standard cultivation conditions.

In general, performed experiments revealed a strong influence of carbon source on biomass and, more importantly, on penitrem biosynthesis. Thus, irrespective to incubation time, *P. crustosum* grown on media without additional carbon supplements was unable to produce detectable amount of penitrems, and also showed almost no growth (Table 1). 

**Figure 5:**
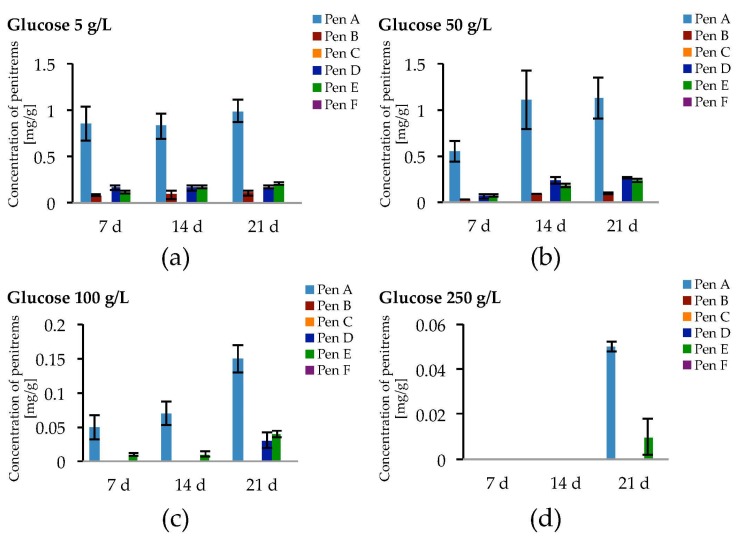
Production of penitrems A–F (mg/g dry mass) after 7, 14 and 21 days by *P. crustosum* cultured on modified CDA supplemented with different concentrations of glucose: 5 g/L (**a**); 50 g/L (**b**); 100 g/L (**c**); and 250 g/L (**d**). Results are mean of five replicates ± standard deviation and given in mg mycotoxin related to g dry mass (mg/g).

Taking the exceptional role of glucose on fungal growth as well as on penitrem production into consideration, other concentrations of glucose were additionally tested (Figure 5 and Figure S3, Table S5). 

Being cultivated on media supplemented with different concentrations of glucose, *P. crustosum* showed more intense growth in media with elevated glucose concentrations (100 g/L and 250 g/L). In contrast, this trend was not observed for penitrem biosynthesis. The increase of the glucose concentration from 5 g/L to 50 g/L was accompanied with a rise of penitrem biosynthesis, whereas higher sugar concentration had an inhibitory effect on the toxin production. Finally, at extreme glucose concentration of 250 g/L, penitrems A and E were only synthesized after a long adaptation period of three weeks and only in low concentrations of 0.05 mg/g and 0.01 mg/g, respectively. The optimum for the production of all penitrems was at 50 g/L and 21 days of incubation.

### 2.3. Impact of the Nitrogen Source on the Production of Penitrems A–F

To study the effect of different nitrogen sources on the penitrem production, media (without yeast extract) were supplemented either with nitrogen containing inorganic salts or with organic compounds such as urea or amino acids (Table 2, Table S6 and S7, Figures S4 and S5). Unlike the case of carbon supplements, where some additives resulted in no penitrem biosynthesis, nitrogen-containing additives, irrespective to nitrogen source, caused production of all penitrems in each experiment. When inorganic salts were used as media supplements, the penitrem production was comparable to those, observed for the standard cultivation conditions reaching, for instance, levels of 1.5–1.8 mg/g for Pen A after 21 days of incubation. No significant differences in the penitrem production were observed in media enriched with various ammonium and nitrate salts or with their combination. With the exception of urea and tryptophan, organic nitrogen sources were found to significantly increase penitrem biosynthesis, when compared to cultivation under standard conditions or grown with nitrogen containing inorganic supplements (Table 2). Among them, glutamate was found to be the most favorable amino acid-based supplement for penitrem production. Thus, *P. crustosum* grown on medium enriched with glutamate (10 mM) produced Pen A in concentration of 4 mg/g as well as measureable amount (ca. 0.2 mg/g) of minor penitrems C and F after 21 days of cultivation. 

**Figure 6:**
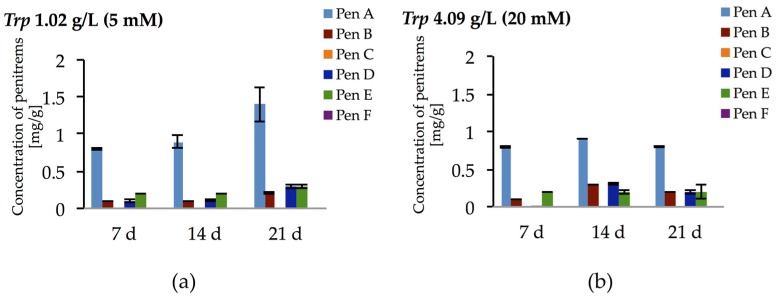
Production of penitrems A–F (mg/g dry mass) after 7, 14 and 21 days by *P. crustosum* cultured on modified CDA supplemented with different concentration of tryptophan: 1.02 g/L (5 mM) (**a**); and 4.09 g/L (20 mM) (**b**). Results are mean of five replicates ± standard deviation and given in mg mycotoxin related to g dry mass (mg/g).

Surprisingly, the tryptophan-rich medium (10 mM) had only inhibitory effects on the production of penitrems (Table 2), despite speculations in the literature suggesting that tryptophan might be directly involved in the biosynthesis of penitrems as a building block for the indole moiety [44]. Therefore, to further elaborate this hypothesis, two more concentrations of tryptophan (5 mM and 20 mM) were tested (Figure 6 and Figure S6, Table S8). However, no dose dependent effect was observed in these experiments with tryptophan. For example, Pen A production after 14 days of cultivation was at the level of 0.9–1.0 mg/g irrespective to content of tryptophan in media. Moreover, the highest tested concentration of tryptophan (20 mM) resulted in decreased accumulation of the toxins, especially after 21 days of incubation. These observations indicate that tryptophan is used by *P. crustosum* as an unspecific nitrogen source rather than as a building block for its direct incorporation into the penitrem structure.

### 2.4. Impact of the Water Activity on the Production of Penitrems A–F

Among environmental factors influencing the fungal growth and mycotoxin production, water activity is known to be one of the most important [45]. Therefore, to evaluate the influence of water activity on the penitrem production, *P. crustosum* was grown on media with adjusted a_w_ values. The water activity was adjusted by addition of different amounts of glycerol with a subsequent determination of the final a_w_ values of resulted media (Table S9, Figure S7). 

**Figure 7:**
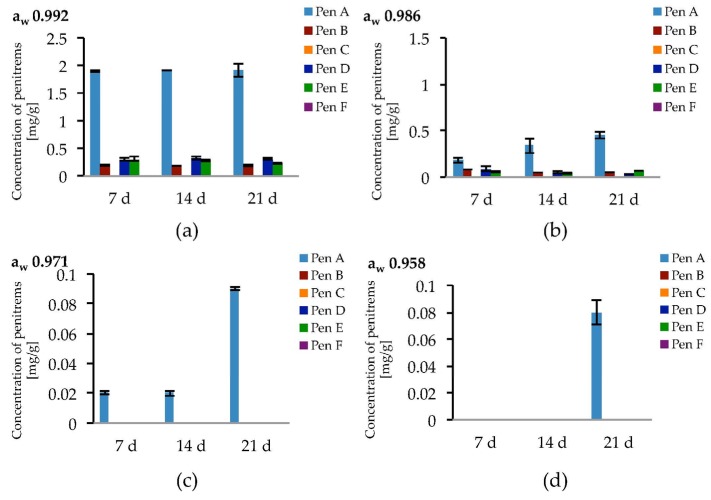
Production of penitrems A–F (mg/g dry mass) after 7, 14 and 21 days by *P. crustosum* cultured on modified CDA supplemented with different concentration of: glycerol 15 g/L (a_w_ 0.992) (**a**); 70 g/L (a_w_ 0.986) (**b**); 200 g/L (a_w_ 0.971) (**c**); and 250 g/L (a_w_ 0.958) (**d**). Results are mean of five replicates ± standard deviation and given in mg mycotoxin related to g dry mass (mg/g).

The highest penitrem levels could be determined in cultures incubated at a_w_ 0.992. However, after seven days of incubation, the concentration of the penitrems (Pen A−1.9 mg/g, Pen B−0.19 mg/g, and Pen D, E−0.3 mg/g) reached a plateau and remained stable for the following two weeks of cultivation (Figure 7a). A decrease of water activity resulted in lower penitrem levels leading to a 2–10 fold decrease at a_w_ 0.986 and the production of only up to 0.9 ng/mL Pen A at a_w_ 0.971 and even less at a_w_ 0.958. Below a_w_ 0.958, *P. crustosum*, still showed fungal growth but no penitrems could be detected (Table S9).

### 2.5. Impact of Salt Stress on the Production of Penitrems A–F

Changes in the osmolarity of the medium can induce so-called salt stress in fungi, significantly influencing the fungal viability as well as the production of secondary metabolites. The osmolarity of media can be adjusted, for instance, by addition of sugar or NaCl. In the present study, there was a particular interest to investigate the effect of salt stress, caused by elevated concentrations of NaCl, on the production of penitrems. Recent reports demonstrate possible relation between NaCl concentration and biosynthesis of ochratoxin A [46]. This toxin, similar to penitrems A, C, and F, represents another example of chlorinated mycotoxins. It was shown that an increased production and excretion of ochratoxin A by Penicillia can be regarded as a kind of adaptation to NaCl-rich environments. The phenomenon of elevated ochratoxin A production by Penicillia under salt stress (NaCl) was attributed to the ability of fungi to pump chlorine out of the cell, ensuring thereby a certain chlorine homeostasis and viability of the Penicillia under these conditions [46]. Therefore, to study whether similar increased biosynthesis of penitrems takes place under salt stress, *P. crustosum* was cultured on media supplemented with different concentration of NaCl (5–100 g/L).

**Figure 8:**
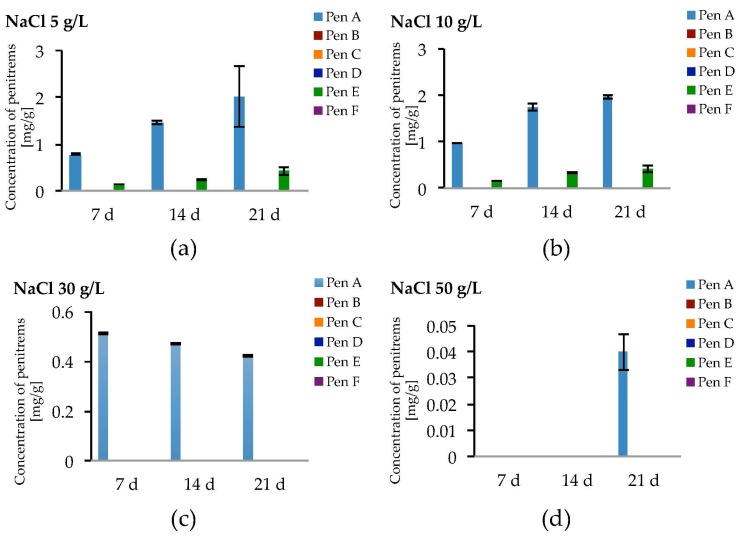
Production of penitrems A–F (mg/g dry mass) after 7, 14 and 21 days by *P. crustosum* cultured on modified CDA supplemented with different concentration of: NaCl 5 g/L (**a**); 10 g/L (**b**); 30 g/L (**c**); and 50 g/L (**d**). Results are mean of five replicates ± standard deviation and given in mg mycotoxin related to g dry mass (mg/g).

Salt stress due to NaCl led to the formation of only Pen A and Pen E in relevant amounts (Figure 8a–d, Table S10). At NaCl levels of 5 g/L and 10 g/L, a slight increase of the production of Pen A compared to the optimized conditions could be observed, though a growth period of three weeks was required and this effect was statistically not significant (Figure 8a,b). Levels of Pen E were comparable to those detected without NaCl while penitrems B, D, C and F could not be detected or have only been found in traces (Figure 8a,b and Figure S8, Table S10). Further elevation of the NaCl concentration to 30 and 50 g/L resulted in substantial decrease of Pen A and Pen E levels. *P. crustosum* grown on medium, supplemented with 50 g/L of NaCl, yielded only 0.04 mg/g of Pen A after 21 days of incubation (Figure 8d).

### 2.6. Impact of the Oxidative Stress on the Production of Penitrems A–F

**Figure 9:**
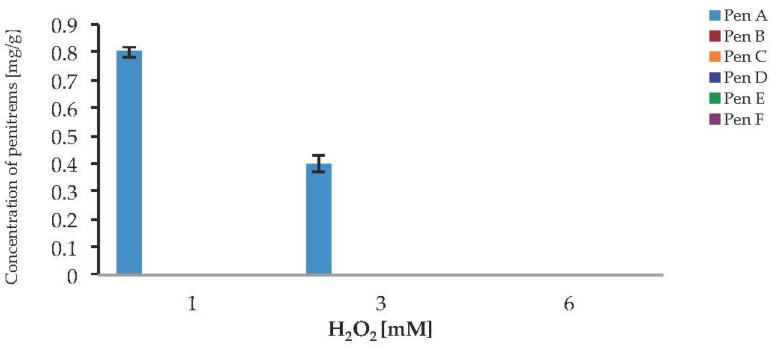
Production of penitrems A–F (mg/g dry mass) after seven days by *P. crustosum* cultured on modified CDA supplemented with different concentration of H_2_O_2_ (0.034 g/L–1 mM, 0.1 g/L–3 mM, and 0.2 g/L–6 mM). Results are mean of five replicates ± standard deviation and given in mg mycotoxin related to g dry mass (mg/g).

To investigate a potential link between oxidative stress and the production of penitrems, the response of *P. crustosum* to the presence of H_2_O_2_ and CuSO_4_ in cultured media was investigated. In the case of H_2_O_2_ no visible growth, and no toxin production was observed, when the fungus was cultivated on media supplemented with 6 mM H_2_O_2_ (Figure 9). The lower levels of H_2_O_2_ allowed the Pen A production after seven days of cultivation, but only in relatively low concentrations of 0.8 mg/g at 1 mM H_2_O_2_ and 0.4 mg/g at 3 mM H_2_O_2_. The Pen A concentration reached its maximum of 0.8 mg/g, when medium was supplemented with 1 mM H_2_O_2_. Irrespective to the studied concentrations of hydrogen peroxide, biosynthesis of other penitrems was completely inhibited. Moreover, starting from day 7 of incubation, inhibiting effect of fungal growth was observed in all tested concentrations of H_2_O_2_, therefore showing no further toxin production (Figure S9). These results demonstrate that H_2_O_2_ has strong inhibitory effect on the *P. crustosum* growth, resulting in undesired loss of the biomass and penitrem production.

**Figure 10:**
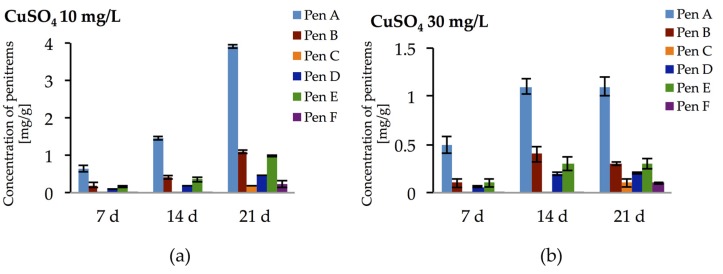
Production of penitrems A–F (mg/g dry mass) after seven days by *P. crustosum* cultured on modified CDA supplemented with different concentration of CuSO_4_: (**a**) 10 mg/L; and (**b**) 30 mg/L. Results are mean of five replicates ± standard deviation and given in mg mycotoxin related to g dry mass (mg/g).

Incubation of *P. crustosum* on medium enriched with CuSO_4_ at 10 and 30 mg/L led to the results summarized in Figure 10. At low CuSO_4_ concentration (10 mg/L), irrespective to incubation period, relatively high amounts of penitrems were produced by *P. crustosum*. Under these conditions already after seven days of incubation, four penitrems (Pen A, B, D, and E) could be detected in quantitable amount (Figure 10a). Further cultivation of *P. crustosum* on medium supplemented with 10 mg/L of CuSO_4_ showed stepwise increase of the concentration of penitrems for 21 days of growth. Thus, after three weeks of incubation concentrations of Pen A, Pen B, and Pen E were at the level of 3.9 mg/g, 1.1 mg/g, and 1.0 mg/g, respectively (Figure 10a), which is twice higher in comparison to production of these toxins by *P. crustosum* grown under standard conditions (Figure 3). Subsequent elevation of CuSO_4_ concentration up to 30 mg/L during the first two weeks of incubation showed a comparable increase of the penitrem levels (Figure 10b). However, between 14 and 21 days of cultivation, no additional increase was observed, though minor members of the family, Pen C and Pen F were produced in concentration of 0.1 mg/g after 21 days of growth (Figure 10b). At a CuSO_4_ concentration of 60 mg/L, no penitrems were detectable in the cultures.

### 2.7. Impact of the Substrate and Food Model Media on the Production of Penitrems A–F 

**Figure 11:**
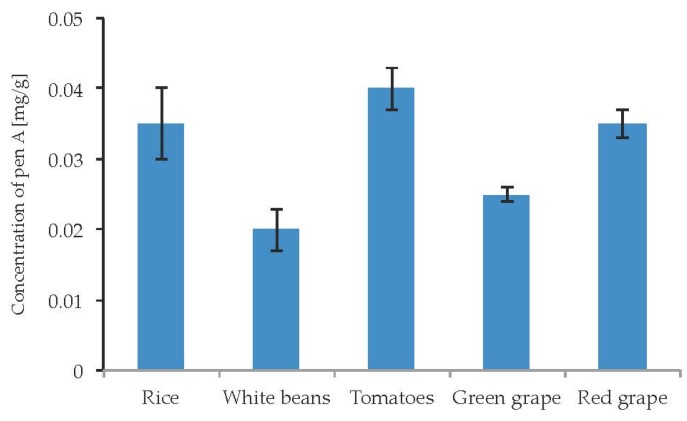
Production of penitrem A (mg/g dry mass) after 14 days by *P. crustosum* cultured on different food substrates at 22 °C. Results are mean of five replicates ± standard deviation and given in mg mycotoxin related to g dry mass (mg/g).

Due to well-documented cases of poisoning by penitrems via the consumption of contaminated food, there was a particular interest to study the influence of different food substrates on the penitrems production. Particularly, several cases of penitrems-associated poisonings were linked to the consumption of contaminated rice, beans, and tomatoes [47]. In addition, Pen A was found to be a contaminant of wine [48]. Rice, white beans, tomatoes as well as green and red grape were inoculated with *P. crustosum* and incubated for two weeks. The cultivation of *P. crustosum* revealed intense fungal growth on all tested food substrates. In addition, irrespective to food source, a production of Pen A was observed in quantitable amounts (Figure 11). No substantial alterations in Pen A biosynthesis were observed on different food substrates. However, rice, tomatoes, and green grape were found to be slightly more favorable for Pen A production, showing the toxin concentrations of 0.02–0.04 mg/g. Other penitrems were not detectable in the samples.

**Figure 12:**
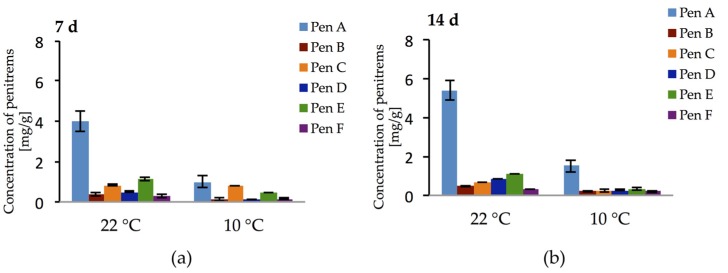
Production of penitrems A–F (mg/g dry mass) after: 7 (**a**); and 14 (**b**) days by *P. crustosum* cultured on cheese model medium. Results are mean of five replicates ± standard deviation and given in mg mycotoxin related to g dry mass (mg/g).

Additionally, the production of penitrems on bread and cheese model media by *P. crustosum* was investigated over a period of two weeks at two different temperatures. In cultures grown on bread model medium, no penitrems could be detected. In contrast, cheese model medium elicited exceptionally high production of all penitrems (Figure 12a,b). Thus, already after the first week of cultivation at 22 °C, Pen A, Pen C, and Pen E were detected at levels of 4 mg/g, 0.83 mg/g, and 1.12 mg/g, respectively. Moreover, after this period, other penitrems were produced in quantitable levels (Figure 12a). Further cultivation of *P. crustosum* on cheese model medium (14 days) led to the increase in Pen A and Pen D production, though no rise in the concentrations of other penitrems was observed (Figure 12b). 

Additionally, to address the question whether penitrems might be produced under lower temperatures on the cheese model medium (imitation of low temperature storage conditions), the experiment was repeated at 10 °C. Interestingly, at 10 °C *P. crustosum* showed not only the ability to grow, though slightly slower, but also to produce all penitrems in quantifiable amounts during two weeks of incubation. (Figure 12a,b).

## 3. Discussion

Penitrems A–F are toxic secondary metabolites which can be produced by several *Penicillium* species. However, the influence of different environmental factors on the biosynthesis of these toxins is not well understood. Therefore, we conducted a detailed study on the impact of different environmental and nutritional factors on the fungal growth as well as the production of penitrems. 

Detailed evaluation of the environmental factors revealed that optimal growth conditions for Pen A production were incubation in the dark on CDA medium with pH close to neutral at 22 °C. CDA medium, compared to other tested media, was found to be the most suitable for Pen A biosynthesis due to its balanced composition, which comprises organic and inorganic nitrogen as well as carbon sources seemingly useful for the toxin production. The relatively low incubation temperature of 22 °C allows *P. crustosum* to grow steadily with the highest level of Pen A accumulation after three weeks. Temperatures below 22 °C slow down the fungal growth, whereas elevated temperatures might lead to faster loss of water. Therefore, in both cases, production of Pen A is reduced but not completely inhibited. These observations are in good agreement with Wagener et al. [34], who reported an increase of Pen A production in cultures of *P. crustosum* grown on YES-medium at 20 °C over 28 days of cultivation. Incubation in the dark, resulted in the fastest biomass production and Pen A accumulation compared to growth under light exposure (day or UV light) where toxin production is decreased, though not completely prevented. A negative influence of light on mycotoxin biosynthesis has been shown for various fungal species [49]. These facts indicate that *P. crustosum* might grow in a wide range of temperatures and under light exposure, producing toxic penitrems and contaminating food under different storing conditions.

Carbon and nitrogen sources are considered to be limiting factors of fungal growth but can also influence the production of secondary metabolites [50,51]. In the course of this study, different mono-, di-, and polysaccharides, as well as amino acids, urea and nitrogen-containing inorganic salts were tested. On complete but reduced media without additional carbon and nitrogen source, *P. crustosum*, showed minimal growth but was unable to produce detectable amount of penitrems. 

Enhanced production of toxins such as ochratoxin, aflatoxin, and tenuazonic acid by different fungi grown on media supplemented with elevated concentrations of monosaccharides was previously reported [52,53,54]. Similarly, in this study, glucose and rhamnose were found to be the most favorable carbon sources for the production of all members of the penitrem family by *P. crustosum.* The optimum for the production of penitrems was found to be at 50 g/L of glucose after 21 days of incubation. Further elevation of the glucose concentration clearly demonstrated an inhibitory effect on the toxin biosynthesis. This effect might be linked to the fact that the high concentration of glucose leads to a decrease of the water activity, which in turns significantly influences the production of penitrems. Thus, when the water activity of the media was gradually decreased from a_w_ 0.995 to a_w_ 0.949, a parallel decrease of the production of penitrem A in cultures of *P. crustosum* was observed. A similar effect was observed during salt stress experiments, when the media were supplemented with different concentrations of NaCl. Thus, media with elevated concentrations of NaCl, more than 50 g/L, were characterized by low a_w_ values, resulting in inhibition of penitrems biosynthesis. This finding is in agreement with a previously reported study, where high relative humidity (99%) was linked to the enhanced production of Pen A [34].

Concerning the organic nitrogen source, generally, amino acids were found to induce the production of all penitrems starting from the first week of incubation. In this study, a particular emphasis was placed on the evaluation of the possible role of tryptophan on the biosynthesis of penitrems, as the first ^14^C-labeling experiments led to the belief that the indole moiety of the indole-diterpenes is originated from tryptophan [44]. Recent studies, however, have indicated that indole-3-glycerol phosphate is the source of indole-diterpenes instead [41]. In addition, in this study, no dose dependent effect was observed in experiment with tryptophan, indicating that tryptophan is used by *P. crustosum* as an unspecific nitrogen source rather than as a building block for its direct incorporation into the penitrem structure. Glutamate instead was found to be an exceptionally favorable amino acid-based supplement for the penitrem production. This finding is in agreement with the indole-3-glycerol phosphate-based pathway of the indole-diterpene biosynthesis, in which the initial step of the chorismat to anthranilate conversion requires glutamine, which in turn is readily produced by fungi from glutamate.

It is reported that oxidative stress caused by the application of blue light as well as by different chemicals can lead to toxin accumulation in fungi. e.g., intracellular oxidative stress was linked to the increased production of aflatoxins in *Aspergillus parasiticus* [55], trichothecenes in *Fusarium* spp. [56], and citrinin in *P. verrucosum* [57]. Overproduction of these secondary metabolites under oxidative stress might be explained by their possible antioxidant and light protective properties. However, to our knowledge nothing is known about the penitrem production by *P. crustosum* under oxidative stress. Therefore, several oxidative stress factors were tested. Hydrogen peroxide was found to be a strong inhibitor of the *P. crustosum* growth, leading to undesired loss of the biomass and decrease in penitrem production. In contrast, biosynthesis of high levels of penitrems was induced by CuSO_4_-promoted oxidative stress (10 mg/L). Among tested abiotic factors, the effect of CuSO_4_ was comparable to that, caused by the elevated concentrations of glutamate.

High incidence of poisonings, caused by the consumption of contaminated food, which is reported to contain high concentration of fungal-born toxins, indicates that food is an exceptionally favorable substrate for toxin production by different filamentous fungi. Thus, for instance, the production of ochratoxin A by *Aspergillus westerdijkiae* is increased when growing on durum wheat semolina compared to cultivation on YES media [58]. In addition, *Alternaria* toxins were reported to be readily produced on synthetic tomato medium [59]. In our study, the cultivation of *P. crustosum* revealed intense fungal growth in all tested food substrates and food model media. However, the conditions, which promote penitrem production, are more complex than those for growth. Although, quantitable concentrations of Pen A were detected in all studied food substrates, quantitation of other penitrems was significantly complicated by matrix effect form extracted food. This effect is highly disadvantageous for the isolation of penitrems, as it also complicates further purification steps. In contrast, cheese model medium elicited the highest production of all members of the penitrems family simultaneously. Cheese medium is rich in organic nitrogen sources and the results described above demonstrate that these compounds are generally supportive for penitrem production. Interestingly, under the lowered temperature (10 °C), *P. crustosum* showed not only the ability to grow on cheese model medium, but was also able to produce all penitrems in quantifiable amount during two weeks of incubation. 

## 4. Conclusions 

The present study gives insights into the production of toxicologically relevant secondary metabolites, namely penitrems A–F, under the combination of different abiotic factors such as medium, incubation time, temperature, pH, light, water activity, and carbon and nitrogen source as well as oxidative and salt stress. For this study, penitrems A–F were isolated from *P. crustosum* cultures and used as analytical standards. Incubation during three weeks in the dark, with pH close to neutral at 22 °C, and a_w_ of 0.99 was found to be optimal conditions for the production of penitrems. Carbon and nitrogen sources were found to be not only limiting factors for the fungal growth, but also for the penitrem production. Thus, among the carbon sources, glucose supplemented to the media at 50 g/L, showed the strongest inducing effect on the biosynthesis of penitrems by *P. crustosum*. Among nitrogen sources, glutamate was found to be the most favorable supplement, significantly increasing production of these secondary metabolites. CuSO_4_-promoted oxidative stress was also shown to remarkably stimulate biosynthesis of all penitrems. In contrast, salt stress induced by elevated NaCl levels, showed an inhibitory effect on the penitrem biosynthesis. In addition, it was noted that the ratio between penitrems remains mostly unchanged, irrespective to the growing condition, when they could be detected in quantitable amount. Cheese model medium elicited exceptionally high production of all penitrems even under the lowered temperature. These findings, on the one hand, indicate perspectives of the use of the cheese model medium as a substrate for the isolation of penitrems as analytical standards. On the other hand, these observations indicate that penitrems could be found in food samples stored under low temperature conditions, signifying thereby the importance of further penitrem studies and their quantitation in food.

## 5. Materials and Methods 

### 5.1. Fungi and Media

*P. crustosum* CBS 483.75 was obtained from the CBS Fungal Biodiversity Centre (an institute of the Royal Netherlands Academy of Arts and Sciences, Utrecht, the Netherlands). In order to obtain pre-cultures 200 mL of liquid media (CD-sucrose (30.0 g/L), NaNO_3_ (2.0 g/L), K_2_HPO_4_ (1.0 g/L), MgSO_4_ × 7H_2_O (0.5 g/L), KCl (0.5 g/L), FeSO_4_x7H_2_O (0.01 g/L), yeast extract (5.0 g/L); ME-malt extract (30.0 g/L), mycological peptone (5 g/L) and YES-yeast extract (5.0 g/L), glucose 30.0 g/L) were autoclaved in 500 mL Erlenmeyer flasks and inoculated with approximately 0.25 cm^2^ of agar plates covered with *P. crustosum*. The flasks were closed with metal caps and incubated using shaking laboratory incubator at 25 °C in the dark. After 3 days *Penicillium* colonies were subcultured onto PDA, CDA, MEA [60] and YES [61] respectively. 

For all experiments, modified Czapek–Dox Agar was used [54]. 

Carbon and nitrogen sources were prepared separately and added after autoclaving. For the nitrogen tests the nitrogen sources ammonium chloride, ammonium sulfate and sodium nitrate of the modified CDA were replaced: NH_4_NO_3_ (0.324 g/L), NaNO_3_ (0.7 g/L), NH_4_Cl + NaNO_3_ (0.12 g/L + 1.0 g/L), (NH_4_)_2_SO_4_ (1.32 g/L) and urea (0.6 g/L). Several amino acids in concentration 10 mM were tested: glycine (0.75 g/L), serine (1.05 g/L), proline (1.15 g/L), arginine (1.74 g/L), and glutamate (1.47 g/L). Tryptophan was added in three different concentrations 1.02, 2.04 and 4.09 g/L. A wide range of different carbon sources in concentration 10 g/L was tested: D-glucose, D-galactose, L-arabinose, D-fructose, D-sucrose, D-xylose, L-rhamnose, D-maltose, cellulose, D-sorbitol and starch.

To analyze the influence of light on the production of penitrems, CDA plates were inoculated and incubated at 22 °C for 3 weeks: in the dark, under the daylight (by using daylight lamp with 380–780 nm) and irradiated with UV (by using UV lamp with 254 nm) light for a certain time course (1 h per each day of experiment). 

The a_w_ in CDA was modified with the respective amount of NaCl (5–100 g/L), glycerol (5–300 g/L) and glucose (5–250 g/L). For incubation at different temperatures (10–34 °C), the inoculated plates were placed in temperature-controlled incubators. The pH was adjusted by adding HCl or NaOH up to the desired value between pH 3 and pH 9 after autoclaving.

To test the influence of an increased oxidative environment on the penitrems, CDA was supplemented with increasing amounts of CuSO_4_ (5–60 mg/L) and H_2_O_2_ (0.034, 0.10, 0.20 g/L).

To investigate the penitrem production on food, 20 g of rice and white beans were transferred in baby food jars with the volume of 200 mL and 7.5 mL of sucrose-casein solution (38 g sucrose and 2.5 g casein were dissolved in 1 L ultra pure water) were added and autoclaved (121 °C for 15 min). Twenty grams of fresh tomatoes and green and red grape were sterilized by treatment with 3% H_2_O_2._ In order to remove H_2_O_2_, all fruits were washed with autoclaved distilled water. Five baby food jars of each food substrates were inoculated with 10 mL of *P. crustosum* liquid culture.

Bread dough was prepared by mixing wheat flour (500 g), margarine (50 g), salt (2.5 g), baking powder (2.5 g), yeast extract (2.5 g), glycerol (50 g) and water (300 mL). 

For cheese model medium 15 g of agar was added to 500 mL of ultra pure water and autoclaved at 121 °C for 15 min. Then 400 g of processed cheese was mixed with warm agar. Portions (20 mL) of the prepared medium were transferred to Petri dishes [62].

### 5.2. Sample Preparation and Determination of Penitrems

To examine the production of penitrems, a slightly modified method of micro-scale extraction developed by Smedsgaard was used [63]. Three plugs (Ø 1.3 cm) from 5 agar plates were cut from the fungal colonies from the region between center and edge of the colony with the aid of sterile corer. The agar plug was transferred to 2 mL Eppendorf^®^ tubes, 1 mL of acetonitrile/water (90/10, *v*/*v*) were added, and the samples were vortexed (VF2 Minishaker, IKA Labortechnik, Staufen, Germany) for 30 s. Subsequent extraction was carried out on a laboratory shaker (New Brunswick, Innova 44, Eppendorf, Wesseling-Berzdorf, Germany) for 25 min at 250 rpm. The supernatant was removed and extraction was repeated. The supernatants were combined and transferred into a vial and evaporated to dryness under a stream of nitrogen at 40 °C. Thereafter, the residue was dissolved in 1 mL of acetonitrile/water (70/30, *v*/*v*) and filtered through a 15 mm syringe filter containing a 0.45 μm pore size RC membrane (Phenomenex, Aschaffenburg, Germany). 

After 7, 14 and 21 days of incubation at 22 °C in the dark, 20 g of the contaminated food was extracted with 80 mL of acetonitrile/water (90/10, *v*/*v*), sonicated for 10 min and shaken on laboratory shaker for 30 min at 200 rpm. The extract was filtered through Miracloth filter material (Merck, Darmstadt, Germany).

The quantitative determination of penitrems was carried out on a Jasco HPLC system (Jasco Labor und Datentechnik, Gross-Umstadt, Germany) equipped with an FLD (FP-1520), DAD (MD-2010 Plus) detectors and autosampler (AS-2057 Jasco). The FLD wavelength was set to excitation at 292 nm and emission at 342 nm. The flow rate was 0.8 mL/min and the injection volume 30 μL. The separation of penitrems was achieved isocratically (acetonitrile/water (70/30, *v*/*v*)) on a Reprosil-Pur, C18-AQ (150 mm, Ø 4 mm i.d., particle size 3 μm) reversed phase column (Dr. Maisch GmbH, Ammerbuch, Germany). Data were collected and analyzed with ChromPass Cromatography (Jasco Labor und Datentechnik, Gross-Umstadt, Germany). Limit of quantification was 0.05 μg/mL for penitrem A, 0.1 μg/mL for penitrem E, 0.2 μg/mL for penitrems D, B, C and F.

Penitrem contents were normalized to biomass and expressed as mg per g dry mass. To determine the dry mass, fungal mycelium from three plugs (Ø 1.3 cm) from five agar plates was transferred in a weighed tube and freeze dried. Additionally fungal mycelium from whole agar plate was freeze dried and weighted. The weight was determined on a standard balance.

### 5.3. Isolation and Analytical Data

For isolation penitrems were extracted with acetonitrile/water (90/10, *v*/*v* from cultures of *P. crustosum* grown on CDA for 3 weeks at 22 °C in the dark. The obtained mixture was purified by NP flash chromatography using a 25 g SNAP cartridge (Biotage, Isolera one, Uppsala, Sweden) with cyclohexane/ethyl acetate gradient. The subsequent separation and purification of penitrems was carried out on a Jasco preparative HPLC system (LC-NetII/ADC Jasco Labor und Datentechnik, Gross-Umstadt, Germany) equipped with UV detector (236 nm). The flow rate was 4 mL/min. The separation of penitrems was carried out on Reprosil-Pur 120 C18-AQ (250 mm, 10 mm i.d., particle size 5 μm) reversed phase column (Dr. Maisch GmbH, Ammerbuch, Germany).

Full set (^1^H, ^13^C, gHMBC, gHSQC and COSY) of NMR spectra of penitrems in CD_3_OD was recorded on an Agilent DD2 600 MHz spectrometer; δ in ppm related to tetramethylsilane (Table S2).

For determination of the exact masses of isolated penitrems a LTQ Orbitrap XL (Thermo Fischer Scientific, Dreieich, Germany) was used with heated electrospray ionization. Sheath gas flow was 5 arbitrary units and aux gas flow 5 arbitrary units. The system was operated in the positive ionization mode with a vaporizer temperature of 300 °C and capillary temperature of 270 °C. The source voltage was 4.1 kV, capillary voltage 5 V and Tube Lens 135 V.

Purity measurements of the penitrems were performed on an HPLC-UV-ELSD with LC-20AT system (Shimadzu, Kyoto, Japan). The column was Reprosil-Pur, C18-AQ (150 mm, 4 mm i.d., particle size 3 μm) (Dr. Maisch GmbH, Ammerbuch, Germany) with acetonitrile as solvent A and water as a solvent B. The wavelength of detector of the UV detector was set to 236 nm, the temperature of the ELSD 40 °C and 2.5 bar of pressurized air were used. 

The gradient of the ELSD measurement starts with 5% A, holding starting condition for 5 min and rising up to 100% A in 25 min followed by a washing step of 5 min with 100% A.

A molar absorptivity value for penitrems in acetonitrile by weighing approximately 3 mg of each compound in a glass flasks were determined. The exact weight was recorded and 10 mL of ACN were added to the flask. The flasks were subsequently placed in an ultrasonic bath for 30 min. UV-vis spectra in a range of 190–400 nm were recorded. The absorptivity value was calculated at the absorption maximum of 292 nm for penitrems A, C, and F, and 286 nm for penitrems B, D, and E using the equation: $\varepsilon =\;\frac{absorption\;x\;1000}{concentration\;in\;mmol/L}$.

All obtained analytical data of isolated penitrems were compared with literature and presented in Supplementary Materials Tables S1 and S2.

## Figures and Tables

**Table 1:** Production of penitrems A–F after 21 days by *P. crustosum* cultured on modified CDA supplemented with different carbon sources.

C Source 10 g/L	Pen A	Pen B	Pen C	Pen D	Pen E	Pen F
None	ND	ND	ND	ND	ND	ND
Glucose	1.13 ± 0.012	0.12 ± 0.037	+	0.26 ± 0.016	0.23 ± 0.033	+
Galactose	0.65 ± 0.013	0.07 ± 0.001	+	0.13 ± 0.002	0.12 ± 0.017	+
Arabinose	0.58 ± 0.072	0.05 ± 0.006	+	0.09 ± 0.001	0.16 ± 0.012	+
Fructose	0.62 ± 0.024	0.06 ± 0.002	+	0.11 ± 0.007	0.12 ± 0.019	+
Sucrose	0.71 ± 0.092	0.07 ± 0.001	+	0.14 ± 0.003	0.11 ± 0.026	+
Xylose	0.54 ± 0.048	0.11 ± 0.001	+	0.11 ± 0.002	0.16 ± 0.008	+
Rhamnose	0.95 ± 0.024	0.13 ± 0.017	+	0.19 ± 0.005	0.19 ± 0.002	+
Maltose	0.76 ± 0.001	0.12 ± 0.062	+	0.19 ± 0.007	0.16 ± 0.003	+
Cellulose	0.34 ± 0.013	+	ND	+	0.07 ± 0.008	ND
Sorbitol	0.45 ± 0.014	0.05 ± 0.011	ND	0.09 ± 0.001	0.08 ± 0.004	ND
Starch	0.28 ± 0.021	+	ND	+	0.05 ± 0.002	ND

Results are mean of five replicates ± standard deviation and given in mg mycotoxin related to g dry mass (mg/g); ND: not determined; + detected with concentration lower than limit of quantitation.

**Table 2:** Production of penitrems A–F after 21 days by *P. crustosum* cultured on modified CDA supplemented with different nitrogen sources.

N Source	Pen A	Pen B	Pen C	Pen D	Pen E	Pen F
None	ND	ND	ND	ND	ND	ND
NaNO_3_	1.5 ± 0.023	0.25 ± 0.004	+	0.31 ± 0.009	0.38 ± 0.008	+
NH_4_NO_3_	1.6 ± 0.055	0.32 ± 0.004	+	0.31 ± 0.080	0.32 ± 0.011	+
(NH_4_)_2_SO_4_	1.6 ± 0.036	0.31 ± 0.047	+	0.22 ± 0.002	0.27 ± 0.092	+
NH_4_Cl+NaNO_3_	1.5 ± 0.075	0.12 ± 0.001	+	0.23 ± 0.009	0.27 ± 0.058	+
NH_4_Cl	1.8 ± 0.026	0.17 ± 0.001	+	0.34 ± 0.006	0.36 ± 0.049	+
Urea	1.1 ± 0.061	0.17 ± 0.005	+	0.17 ± 0.008	0.24 ± 0.001	+
*Gly*	2.0 ± 0.073	0.30 ± 0.006	+	0.35 ± 0.004	0.32 ± 0.002	+
*Ser*	2.0 ± 0.089	0.42 ± 0.009	+	0.46 ± 0.069	0.51 ± 0.001	+
*Pro*	2.1 ± 0.062	0.36 ± 0.019	+	0.37 ± 0.023	0.42 ± 0.008	+
*Arg*	1.9 ± 0.089	0.31 ± 0.029	+	0.34 ± 0.044	0.35 ± 0.016	+
*Glu*	4.0 ± 0.284	0.79 ± 0.031	0.18 ± 0.053	0.85 ± 0.022	0.91 ± 0.003	0.15 ± 0.017
*Trp*	0.92 ± 0.052	0.28 ± 0.020	+	0.32 ± 0.046	0.38 ± 0.001	+

Results are mean five replicates ± standard deviation and given in mg mycotoxin related to g dry mass (mg/g); ND: not determined; + detected with concentration lower than limit of quantitation.

## Supplementary Materials

The following are available online at www.mdpi.com/2072-6651/9/7/210/s1, Table S1: Analytical data of isolated penitrems A–F, Table S2: NMR spectra of isolated penitrems A–F, Table S3: Penitrems production after 7, 14 and 21 days by *P. crustosum* in Czapek–Dox Agar in the dark at 22 °C (standard conditions), Table S4: Penitrems production after 7, 14 and 21 days by *P. crustosum* in modified Czapek–Dox media supplemented with different carbon sources, Table S5: Penitrems production after 7, 14 and 21 days by *P. crustosum* in modified Czapek–Dox media supplemented with glucose, Table S6: Penitrems production after 7, 14 and 21 days by *P. crustosum* in modified Czapek–Dox media supplemented with different inorganic nitrogen sources, Table S7: Penitrems production after 7, 14 and 21 days by *P. crustosum* in modified Czapek–Dox media supplemented with different organic nitrogen sources, Table S8: Penitrems production after 7, 14 and 21 days by *P. crustosum* in modified Czapek–Dox media supplemented with tryptophan, Table S9: Penitrems production after 7, 14 and 21 days by *P. crustosum* in modified Czapek–Dox media supplemented with glycerol, Table S10: Penitrems production after 7, 14 and 21 days by *P. crustosum* in modified Czapek–Dox media supplemented with NaCl, Figure S1: Effect of: media (a); temperature (b); pH (c); and light (d) on *P. crustosum* growth after 7, 14 and 21 days of incubation, Figure S2: The growth of *P. crustosum* cultured on modified CDA supplemented with different C sources after 7, 14 and 21 days of incubation, Figure S3: The growth of *P. crustosum* cultured on modified CDA supplemented with different concentration of glucose after 7, 14 and 21 days of incubation, Figure S4: The growth of *P. crustosum* cultured on modified CDA supplemented with different inorganic nitrogen sources after 7, 14 and 21 days of incubation, Figure S5: The growth of *P. crustosum* cultured on modified CDA supplemented with different organic nitrogen sources after 7, 14 and 21 days of incubation, Figure S6: The growth of *P. crustosum* cultured on modified CDA supplemented with different concentration of tryptophan after 7, 14 and 21 days of incubation, Figure S7: The growth of *P. crustosum* cultured on modified CDA supplemented with different concentration of glycerol after 7, 14 and 21 days of incubation, Figure S8: The growth of *P. crustosum* cultured on modified CDA supplemented with different concentration of NaCl after 7, 14 and 21 days of incubation, Figure S9: The growth of *P. crustosum* cultured on modified CDA supplemented with different concentration of H_2_O_2_ after 7, 14 and 21 days of incubation.

## Abbreviations

GABA	gamma-aminobutyric acid
BK channels	big potassium channels, also called Maxi-K or slo1
CNS	central nervous system
BBB	blood brain barrier
HPLC	high performance liquid chromatography
YES	yeast extract sucrose medium
CDA	Czapek–Dox Agar
MEA	malt extract agar
Pen	penitrem
a_w_	water activity
NP	normal phase
