# Supplementary material for: Influence of Environmental Factors on the Production of Penitrems A–F by Penicillium crustosum
Source: Toxins (Basel). 2017 Jul 1;9(7):210. doi: 10.3390/toxins9070210 (PMC5535157; doi:10.3390/toxins9070210)
Supplement: Supplementary file 1 [file toxins-09-00210-s001.pdf]

Supplementary Materials: Influence of environmental factors on the production of penitrems A–F by *Penicillium crustosum*

Svetlana A. Kalinina, Annika Jagels, Benedikt Cramer, Rolf Geisen and Hans-Ulrich Humpf

Table S1. Analytical data of isolated penitrems (A–F).

Name of Uhhcompound	Molecular Formula	Appearance	Yield (mg)	Purity, % (HPLC-UV-ELSD)	UV λ^{ACN}_{max}, nm	Exact Mass $(M + H)^+$
Pen A	$C_{37}H_{44}NO_6Cl$	Colorless amorphous solid	14.4	99	232 and 296, ε = 11.500	634.29138
Pen B	$C_{37}H_{45}NO_5$	Colorless amorphous solid	7.4	96	220 and 288, ε = 12.000	584.33569
Pen C	$C_{37}H_{44}NO_4Cl$	Colorless amorphous solid	3.5	96	232 and 296, ε = 12.700	602.30188
Pen D	$C_{37}H_{45}NO_4$	Colorless amorphous solid	6.5	97	220 and 288, ε = 11.100	568.34192
Pen E	$C_{37}H_{45}NO_6$	Colorless amorphous solid	11.4	98	220 and 288, ε = 11.100	600.33081
Pen F	$C_{37}H_{44}NO_5Cl$	Colorless amorphous solid	6.5	96	232 and 296, ε = 11.100	618.29749

Table S2. NMR spectra of isolated penitrems (A–F).

^{1}H NMR (CD_3OD)	^{13}C NMR (CD_3OD)
**Pen A**	
δ [ppm] = 1.11 (s, 3H),1.27 (s, 3H), 1.39 (s, 3H), 1.45–1.52 (m, 1H), 1.57–1.64 (m, 1H), 1.72 (s, 3H), 1.73 (s, 3H), 1.74–1.80 (m, 2H), 1.88–1.97 (m, 1H), 2.07–2.21 (m, 1H), 2.22–2.33 (m, 2H), 2.39 (td, *J* = 11.3, 9.4 Hz, 1H), 2.49–2.55 (m, 1H), 2.61 (td, *J* = 13.6, 5.3 Hz, 1H), 2.66 (ddd, *J* = 13.2, 8.2, 2.5 Hz, 1H), 2.91–2.97 (m, 1H), 3.28–3.30 (m, 1H), 3.50 (dd, *J* = 3.1, 1.4 Hz, 1H), 3.68 (d, *J* = 15.9 Hz, 1H), 4.03–4.08 (m, 2H), 4.30 (ddd, *J* = 9.6, 8.0, 1.2 Hz, 1H), 4.86–4.88 (m, 1H), 4.91–4.93 (m, 1H), 4.95 (d, *J* = 8.2 Hz, 1H), 5.00–5.03 (m, 1H), 5.09–5.11 (m, 1H), 7.24 (s, 1H).	δ [ppm] = 18.9 (1C, C-20), 19.1 (1C, C-39), 19.8 (1C, C-36), 20.3 (1C, C-34), 21.2 (1C, C-40), 25.0 (1C, C-13), 27.0 (1C, C-30), 29.1 (1C, C-29), 30.65 (1C, C-35), 30.68 (1C, C-21), 35.4 (1C, C-10), 44.0 (1C, C-31), 47.3 (1C, C-12), 50.6 (1C, C-32), 53.3 (1C, C-14), 59.0 (1C, C-19), 62.2 (1C, C-24), 66.5 (1C, C-25), 66.7 (1C, C-23), 72.7 (1C, C-28), 73.6 (1C, C-18), 75.1 (1C, C-26), 77.4 (1C, C-16), 78.6 (1C, C-22), 81.5 (1C, C-15), 107.2 (1C, C-33), 111.9 (1C, C-38), 112.2 (1C, C-7), 119.8 (1C, C-3), 121.8 (1C, C-8), 125.3 (1C, C-6), 126.3 (1C, C-5), 132.8 (1C, C-4), 140.2 (1C, C-9), 143.2 (1C, C-37), 149.7 (1C, C-11), 155.0 (1C, C-2).
**Pen B**	
δ [ppm] = 1.15 (s, 3H), 1.25 (s, 3H), 1.37 (s, 3H), 1.44–1.51 (m, 1H), 1.55 (s, 3H), 1.60–1.65 (m, 1H), 1.69–1.79 (m, 2H), 1.72 (s, 3H), 1.87–1.95 (m, 1H), 2.09–2.22 (m, 3H), 2.25–2.33 (m, 2H), 2.42–2.49 (m, 1H), 2.56–2.67 (m, 2H), 3.07–3.14 (m, 1H), 3.27 (d, *J* = 15.1 Hz, 1H), 3.43 (d, *J* = 15.4 Hz, 1H), 3.50 (dd, *J* = 3.2, 1.4 Hz, 1H), 3.86 (t, *J* = 9.6 Hz, 1H), 4.03–4.07 (m, 2H), 4.29 (t, *J* = 8.9 Hz, 1H), 4.81 (d, *J* = 8.1 Hz, 1H), 4.90–4.93 (m, 1H), 5.02–5.05 (m, 1H), 5.08–5.12 (m, 1H), 6.71 (d, *J* = 8.2 Hz, 1H), 7.04 (dd, *J* = 8.2, 0.7 Hz, 1H).	δ [ppm] = 18.8 (1C, C-20), 18.9 (1C, C-39), 19.0 (1C, C-34), 19.8 (1C, C-36), 21.2 (1C, C-40), 27.0 (1C, C-13), 27.1 (1C, C-30), 28.5 (1C, C-35), 29.1 (1C, C-29), 30.6 (1C, C-21), 35.5 (1C, C-12), 39.0 (1C, C-10), 39.8 (1C, C-15), 44.1 (1C, C-31), 50.3 (1C, C-32), 52.9 (1C, C-14), 59.3 (1C, C-19), 62.3 (1C, C-24), 66.5 (1C, C-23), 66.7 (1C, C-25), 72.8 (1C, C-28), 73.3 (1C, C-18), 75.1 (1C, C-26), 76.9 (1C, C-16), 78.6 (1C, C-22), 106.1 (1C, C-33), 110.5 (1C, C-7), 111.9 (1C, C-38), 118.7 (1C, C-3), 121.3 (1C, C-6), 123.1 (1C, C-8), 128.5 (1C, C-5), 128.7 (1C, C-4), 143.2 (1C, C-37), 150.5 (1C, C-11), 139.7 (1C, C-9), 153.7 (1C, C-2).
**Pen C**	
δ [ppm] = 1.11 (s, 3H), 1.21 (s, 3H), 1.42 (s, 3H), 1.56 (s, 3H), 1.62–1.66 (m, 1H), 1.71–1.76 (m, 1H), 1.79 (s, 3H), 1.82–1.88 (m, 1H), 1.89–2.00 (m, 2H), 2.01–2.08 (m, 1H), 2.10–2.23 (m, 2H), 2.27–2.34 (m, 1H), 2.42–3.50 (m, 1H), 2.60 (td, *J* = 13.6, 4.8 Hz, 1H), 2.65 (ddd, *J* = 13.3, 8.0, 2.5 Hz, 1H), 3.07–3.14 (m, 1H), 3.26 (d, *J* = 15.1 Hz, 1H), 3.43 (d, J = 14.9 Hz, 1H), 3.83–3.89 (m, 2H), 3.9 –3.98 (m, 1H), 4.58–4.63 (m, 1H), 4.82–4.84 (m, 2H), 4.92–4.96 (m, 1H), 5.02–5.05 (m, 1H), 5.11–5.15 (m, 1H), 5.73 (dd, *J* = 5.9, 1.9 Hz, 1H), 7.03 (d, *J* = 8.2 Hz, 1H).	δ [ppm] = 18.8 (1C, C-34), 19.4 (1C, C-20), 20.0 (1C, C-36), 20.2 (1C, C-39), 21.3 (1C, C-40), 27.1 (1C, C-13), 27.9 (1C, C-30), 28.4 (1C, C-35), 29.4 (1C, C-29), 34.9 (1C, C-12), 35.5 (1C, C-21), 39.2 (1C, C-10), 39.8 (1C, C-15), 44.0 (1C, C-31), 50.6 (1C, C-32), 52.9 (1C, C-14), 59.0 (1C, C-19), 64.5 (1C, C-25), 73.1 (1C, C-18), 75.2 (1C, C-26), 76.9 (1C, C-16), 77.6 (1C, C-22), 80.8 (1C, C-28), 106.0 (1C, C-33), 110.5 (1C, C-7), 111.4 (1C, C-38), 118.4 (1C, C-3), 118.9 (1C, C-24), 121.2 (1C, C-6), 123.3 (1C, C-8), 128.5 (1C, C-5), 128.7 (1C, C-4), 139.8 (1C, C-9), 143.6 (1C, C-37), 149.5 (1C, C-23), 148.8 (1C, C-11), 154.2 (1C, C-2).
**Pen D**	
δ [ppm] = 1.09 (s, 3H), 1.16 (s, 3H), 1.41 (s, 3H), 1.56 (s, 3H), 1.62–1.66 (m, 1H), 1.71–1.76 (m, 1H), 1.79 (s, 3H), 1.82–1.88 (m, 1H), 1.89–2.00 (m, 2H), 2.01–2.08 (m, 1H), 2.10–2.23 (m, 2H), 2.27–2.34 (m, 1H), 2.42–3.50 (m, 1H), 2.60 (td, *J* = 13.6, 4.8 Hz, 1H), 2.65 (ddd, *J* = 13.3, 8.0, 2.5 Hz, 1H), 3.07–3.14 (m, 1H), 3.26 (d, *J* = 15.1 Hz, 1H), 3.43 (d, J = 14.9 Hz, 1H), 3.83–3.89 (m, 2H), 3.95–3.98 (m, 1H), 4.58–4.63 (m, 1H), 4.82–4.84 (m, 2H), 4.92–4.96 (m, 1H), 5.02–5.05 (m, 1H), 5.11–5.15 (m, 1H), 5.71 (dd, *J* = 5.9, 1.9 Hz, 1H), 6.71 (d, *J* = 8.3 Hz, 1H), 7.03 (d, *J* = 8.2 Hz, 1H).	δ [ppm] = 18.8 (1C, C-34), 19.4 (1C, C-20), 20.0 (1C, C-36), 20.2 (1C, C-39), 21.3 (1C, C-40), 27.1 (1C, C-13), 27.8 (1C, C-30), 28.5 (1C, C-35), 29.4 (1C, C-29), 35.1 (1C, C-12), 35.5 (1C, C-21), 39.0 (1C, C-10), 39.8 (1C, C-15), 44.1 (1C, C-31), 50.4 (1C, C-32), 52.9 (1C, C-14), 59.0 (1C, C-19), 64.5 (1C, C-25), 73.4 (1C, C-18), 75.2 (1C, C-26), 76.9 (1C, C-16), 77.7 (1C, C-22), 80.8 (1C, C-28), 106.0 (1C, C-33), 110.5 (1C, C-7), 111.4 (1C, C-38), 118.4 (1C, C-3), 118.8 (1C, C-24), 121.2 (1C, C-6), 123.1 (1C, C-8), 128.5 (1C, C-5), 128.7 (1C, C-4), 139.7 (1C, C-9), 143.6 (1C, C-37), 149.6 (1C, C-23), 150.5 (1C, C-11), 154.2 (1C, C-2).
**Pen E**	
δ [ppm] = 1.10 (s, 3H), 1.28 (s, 3H), 1.40 (s, 3H), 1.46–1.53 (m, 1H), 1.61–1.66 (m, 1H), 1.72 (s, 3H), 1.73 (s, 3H), 1.74–1.81 (m, 2H), 1.89–1.97 (m, 1H), 2.09–2.16 (m, 1H), 2.24 (ddd, *J* = 11.7, 9.3, 2.7 Hz, 1H), 2.27–2.32 (m, 1H), 2.35–2.42 (m, 1H), 2.50–2.55 (m, 1H), 2.61 (td, *J* = 13.6, 5.3 Hz, 1H), 2.67 (ddd, *J* = 13.1, 8.2, 2.6 Hz, 1H), 2.92–2.97 (m, 1H), 3.25 (d, *J* = 15.0 Hz, 1H), 3.49–3.52 (m, 1H), 3.55 (d, *J* = 15.1 Hz, 1H), 4.03–4.07 (m, 2H), 4.30 (t, *J* = 8.9 Hz, 1H), 4.79–4.81 (m, 1H), 4.90–4.93 (m, 1H), 4.94–4.96 (m, 1H), 4.97 (d, *J* = 8.1 Hz, 1H), 5.08–5.12 (m, 1H), 6.74 (d, *J* = 8.2 Hz, 1H), 7.12 (d, *J* = 8.2 Hz, 1H).	δ [ppm] = 18.9 (1C, C-20), 19.1 (1C, C-39), 19.8 (1C, C-36), 20.3 (1C, C-34), 21.3 (1C, C-40), 25.0 (1C, C-13), 27.0 (1C, C-30), 29.1 (1C, C-29), 30.6 (1C, C-35), 30.7 (1C, C-21), 38.4 (1C, C-10), 44.1 (1C, C-31), 47.7 (1C, C-12), 50.5 (1C, C-32), 53.5 (1C, C-14), 59.0 (1C, C-19), 62.3 (1C, C-24), 66.5 (1C, C-25), 66.8 (1C, C-23), 72.8 (1C, C-28), 73.8 (1C, C-18), 75.1 (1C, C-26), 77.5 (1C, C-16), 78.6 (1C, C-22), 81.8 (1C, C-15), 105.7 (1C, C-33), 111.9 (1C, C-7), 112.1 (1C, C-38), 119.5 (1C, C-3), 120.8 (1C, C-6), 122.5 (1C, C-8), 128.7 (1C, C-5), 131.0 (1C, C-4), 140.7 (1C, C-9), 143.2 (1C, C-37), 150.9 (1C, C-11), 154.2 (1C, C-2).
**Pen F**	
δ [ppm] = 1.15 (s, 3H), 1.24 (s, 3H), 1.37 (s, 3H), 1.43–1.50 (m, 1H), 1.55 (s, 3H), 1.57–1.63 (m, 1H), 1.72 (s, 3H), 1.73–1.81 (m, 2H), 2.00–2.17 (m, 2H), 2.17–2.24 (m, 1H), 2.25–2.34 (m, 1H), 2.44–3.51 (m, 1H), 2.56–2.66 (m, 2H), 2.78 (t, *J* = 6.9 Hz, 1H), 3.18–3.21 (m, 1H), 3.48–3.51 (m, 1H), 3.70 (d, *J* = 15.9 Hz, 1H), 3.86 (t, *J* = 9.7 Hz, 1H), 4.02–4.07 (m, 2H), 4.29 (t, *J* = 8.8 Hz, 1H), 4.80 (d, *J* = 8.1 Hz, 1H), 4.88–4.93 (m, 3H), 5.08–5.12 (m, 2H), 7.16 (s, 1H).	δ [ppm] = 18.8 (1C, C-20), 19.0 (1C, C-34), 19.8 (1C, C-36), 21.1 (1C, C-40), 26.5 (1C, C-13), 27.0 (1C, C-30), 28.5 (1C, C-35), 29.0 (1C, C-29), 30.8 (1C, C-21), 34.4 (1C, C-12), 35.3 (1C, C-10), 40.0 (1C, C-15), 44.0 (1C, C-31), 50.4 (1C, C-32), 52.9 (1C, C-14), 59.3 (1C, C-19), 62.2 (1C, C-24), 66.5 (1C, C-25), 72.1 (2C, C-23, C-28), 72.1 (1C, C-18), 75.1 (1C, C-26), 76.9 (1C, C-16), 77.0 (1C, C-22), 107.4 (1C, C-33), 110.8 (1C, C-7), 111.4 (1C, C-38), 129.1 (1C, C-4), 137.0 (1C, C-9), 143.3 (1C, C-37), signals of C-2, C-3, C-5, C-6, C-8, and C-11 could not be observed on the spectrum.

Table S3. Penitrems production after 7, 14 and 21 days by *P. crustosum* in Czapek-Dox Agar in the dark at 22 °C (standard conditions).

Days	Pen A		Pen B		Pen C		Pen D		Pen E		Pen F	
	μg/mL	mg/g	μg/mL	mg/g	μg/mL	mg/g	μg/mL	mg/g	μg/mL	mg/g	μg/mL	mg/g
7	9.82 ± 0.25	0.5 ± 0.04	+		ND		+		1.96 ± 0.12	0.12 ± 0.04	ND	
14	21.12 ± 0.53	1.1 ± 0.08	5.43 ± 1.23	0.27 ± 0.09	+		5.41 ± 0.95	0.27 ± 0.02	5.63 ± 1.78	0.28 ± 0.09	+	
21	32.23 ± 1.23	1.6 ± 0.02	8.25 ± 1.54	0.41 ± 0.01	1.20 ± 0.12	0.06 ± 0.01	7.65 ± 1.23	0.38 ± 0.05	10.27 ± 1.98	0.51 ± 0.07	1.20 ± 0.76	0.06 ± 0.01

Results are mean five replicates ± standard deviation and given in μg mycotoxin per milliliter (μg/mL) and mg mycotoxin related to g dry mass (mg/g); ND: not detected; + detected with concentration lower than limit of quantitation.

Table S4. Penitrems production after 7, 14 and 21 days by *P. crustosum* in modified Czapek-Dox media supplemented with different carbon sources.

C source	Days	Pen A		Pen B		Pen C		Pen D		Pen E		Pen F	
		µg/mL	mg/g	µg/mL	mg/g	µg/mL	mg/g	µg/mL	mg/g	µg/mL	mg/g	µg/mL	mg/g
None added	7,14,21	ND		ND		ND		ND		ND		ND	
Glucose	7	14.21 ± 0.21	0.99 ± 0.051	2.58 ± 0.11	0.18 ± 0.005	+		2.59 ± 0.11	0.18 ± 0.071	2.58 ± 0.37	0.18 ± 0.002	+	
	14	16.21 ± 0.48	0.92 ± 0.021	2.11 ± 0.82	0.12 ± 0.106	+		3.70 ± 0.71	0.21 ± 0.013	3.17 ± 0.21	0.18 ± 0.001	+	
	21	22.38 ± 2.92	1.13 ± 0.012	2.38 ± 0.27	0.12 ± 0.037	+		5.15 ± 0.36	0.26 ± 0.016	4.55 ± 0.92	0.23 ± 0.033	+	
Galactose	7	10.11 ± 0.61	0.51 ± 0.014	0.99 ± 0.11	0.05 ± 0.001	ND		1.78 ± 0.14	0.09 ± 0.001	2.37 ± 0.44	0.12 ± 0.004	ND	
	14	12.32 ± 0.13	0.54 ± 0.024	1.59 ± 0.17	0.07 ± 0.023	+		3.19 ± 0.27	0.14 ± 0.091	2.51 ± 0,69	0.11 ± 0.061	+	
	21	15.32 ± 0.19	0.65 ± 0.013	1.65 ± 0.13	0.07 ± 0.001	+		3.15 ± 0.18	0.13 ± 0.002	2.82 ± 0.16	0.12 ± 0.017	+	
Arabinose	7	9.81 ± 0.53	0.46 ± 0.006	0.43+0.11	0.02 ± 0.001	ND		1.11 ± 0.12	0.05 ± 0.001	1.71 ± 0.11	0.08 ± 0.003	ND	
	14	10.42 ± 2.75	0.46 ± 0.042	1.13 ± 0.37	0.05 ± 0.001	+		2.04 ± 0.18	0.09 ± 0.082	1.81 ± 0.37	0.08 ± 0.002	+	
	21	14.38 ± 0.62	0.58 ± 0.072	1.24 ± 0.26	0.05 ± 0.006	+		2.23 ± 0.11	0.09 ± 0.001	3.97 ± 0.67	0.16 ± 0.012	+	
Fructose	7	9.89 ± 1.19	0.46 ± 0.003	1.08 ± 0.51	0.05 ± 0.001	ND		1.08 ± 0.49	0.05 ± 0.017	1.72 ± 0.82	0.08 ± 0.001	ND	
	14	14.21 ± 0.93	0.52 ± 0.028	1.37 ± 0.36	0.05 ± 0.001	+		3.01 ± 0.41	0.11 ± 0.081	3.01 ± 0.11	0.11 ± 0.004	+	
	21	17.83 ± 0.32	0.62 ± 0.024	1.72 ± 0.31	0.06 ± 0.002	+		3.16 ± 0.84	0.11 ± 0.007	3.44 ± 0.72	0.12 ± 0.019	+	
Sucrose	7	8.31 ± 1.27	0.41 ± 0.001	0.81 ± 0.13	0.04 ± 0.019	+		3.05 ± 0.11	0.15 ± 0.069	1.62 ± 0.45	0.08 ± 0.001	+	
	14	12.67 ± 0.82	0.61 ± 0.661	1.25 ± 0.14	0.06 ± 0.009	+		2.28 ± 0.12	0.11 ± 0.024	2.28 ± 0.12	0.11 ± 0.121	+	
	21	15.23 ± 0.92	0.71 ± 0.092	1.51 ± 0.12	0.07 ± 0.001	+		3.01 ± 0.52	0.14 ± 0.003	2.36 ± 0.66	0.11 ± 0.026	+	
Xylose	7	11.23 ± 0.71	0.45 ± 0.007	0.99 ± 0.11	0.04 ± 0.041	ND		1.99 ± 0.11	0.08 ± 0.004	2.25 ± 0.56	0.09 ± 0.004	ND	
	14	13.91 ± 0.56	0.55 ± 0.091	2.78 ± 0.31	0.11 ± 0.008	ND		2.78 ± 0.16	0.11 ± 0.001	1.93 ± 0.12	0.09 ± 0.001	ND	
	21	15.31 ± 0.15	0.54 ± 0.048	3.12 ± 0.53	0.11 ± 0.001	+		3.12 ± 0.74	0.11 ± 0.002	4.54 ± 0.83	0.16 ± 0.008	+	
Rhamnose	7	9.62 ± 0.13	0.51 ± 0.032	0.19 ± 0.22	0.01 ± 0.001	ND		1.32 ± 0.11	0.07 ± 0.002	1.51 ± 0.77	0.08 ± 0.001	ND	
	14	15.34 ± 0.11	0.77 ± 0.113	1.39 ± 0.67	0.07 ± 0.002	+		2.79 ± 0.32	0.14 ± 0.004	2.99 ± 0.73	0.15 ± 0.004	+	
	21	19.27 ± 0.42	0.95 ± 0.024	2.64 ± 0.17	0.13 ± 0.017	+		3.85 ± 0.91	0.19 ± 0.005	3.85 ± 0.17	0.19 ± 0.002	+	
Maltose	7	10.11 ± 0.31	0.41 ± 0.014	0.49 ± 0.11	0.02 ± 0.001	ND		1.97 ± 0.12	0.08 ± 0.004	1.97 ± 0.11	0.08 ± 0.001	ND	
	14	15.29 ± 1.32	0.62 ± 0.021	1.97 ± 0.11	0.08 ± 0.093	+		3.95 ± 0.82	0.16 ± 0.011	3.45 ± 0.73	0.14 ± 0.001	+	
	21	19.81 ± 0.78	0.76 ± 0.001	3.13 ± 0.42	0.12 ± 0.062	+		4.95 ± 0.78	0.19 ± 0.007	4.17 ± 0.21	0.16 ± 0.003	+	
Cellulose	7	4.98 ± 0.31	0.17 ± 0.075	+		ND		+		0.88 ± 0.14	0.03 ± 0.001	ND	
	14	9.23 ± 0.65	0.31 ± 0.029	+		ND		+		1.48 ± 0.91	0.05 ± 0.001	ND	
	21	10.21 ± 0.94	0.34 ± 0.013	+		ND		+		2.11 ± 0.29	0.07 ± 0.008	ND	
Sorbitol	7	6.97 ± 0.43	0.22 ± 0.103	0.95 ± 0.11	0.03 ± 0.061	ND		0.95 ± 0.23	0.03 ± 0.001	1.27 ± 0.11	0.04 ± 0.001	ND	
	14	10.18 ± 0.23	0.31 ± 0.059	1.64 ± 0.28	0.05 ± 0.083	ND		2.29 ± 0.12	0.07 ± 0.003	1.64 ± 0.18	0.05 ± 0.001	ND	
	21	15.13 ± 0.51	0.45 ± 0.014	1.68 ± 0.11	0.05 ± 0.011	ND		3.02 ± 0.11	0.09 ± 0.001	2.69 ± 0.31	0.08 ± 0.004	ND	
Starch	7	5.21 ± 0.71	0.16 ± 0.091	+		ND		+		0.98 ± 0.11	0.03 ± 0.001	ND	
	14	10.14 ± 0.12	0.31 ± 0.084	+		ND		+		1.68 ± 0.11	0.05 ± 0.047	ND	
	21	11.92 ± 0.79	0.28 ± 0.021	+		ND		+		2.12 ± 0.12	0.05 ± 0.002	ND	

Results are mean five replicates ± standard deviation and given in µg mycotoxin per milliliter (µg/mL) and mg mycotoxin related to g dry mass (mg/g); ND: not detected; + detected with concentration lower than limit of quantitation.

Table S5. Penitrems production after 7, 14 and 21 days by *P. crustosum* in modified Czapek-Dox media supplemented with glucose.

a_w	Concentration of glucose (g/L)	Days	Pen A		Pen B		Pen C		Pen D		Pen E		Pen F	
			µg/mL	mg/g	µg/mL	mg/g	µg/mL	mg/g	µg/mL	mg/g	µg/mL	mg/g	µg/mL	mg/g
0.993	5	7	16.45 ± 2.04	0.85 ± 0.18	1.54 ± 0.18	0.08 ± 0.012	+		3.11 ± 0.66	0.16 ± 0.021	2.32 ± 0.31	0.12 ± 0.019	+	
		14	18.12 ± 1.47	0.83 ± 0.14	1.96 ± 0.81	0.09 ± 0.053	+		3.49 ± 0.21	0.16 ± 0.035	3.71 ± 0.11	0.17 ± 0.011	+	
		21	21.78 ± 1.77	0.99 ± 0.12	2.42 ± 0.17	0.11 ± 0.031	+		3.74 ± 0.29	0.17 ± 0.011	4.62 ± 0.35	0.21 ± 0.016	+	
0.989	50	7	12.78 ± 1.51	0.55 ± 0.11	0.71 ± 0.021	0.03 ± 0.004	+		1.39 ± 0.49	0.06 ± 0.021	1.63 ± 0.25	0.07 ± 0.011	+	
		14	25.79 ± 0.91	1.11 ± 0.32	2.09 ± 0.071	0.09 ± 0.001	+		5.34 ± 0.12	0.23 ± 0.039	4.18 ± 0.12	0.18 ± 0.025	+	
		21	28.78 ± 4.55	1.13 ± 0.23	2.29 ± 0.15	0.09 ± 0.009	+		6.88 ± 0.12	0.27 ± 0.012	5.86 ± 0.25	0.23 ± 0.017	+	
0.986	100	7	2.86 ± 0.14	0.05 ± 0.018	+		+		+		0.57 ± 0.13	0.01 ± 0.002	+	
		14	4.13 ± 0.25	0.07 ± 0.017	+		+		+		0.59 ± 0.028	0.01 ± 0.004	+	
		21	9.52 ± 0.51	0.15 ± 0.021	+		+		1.91 ± 0.14	0.03 ± 0.011	2.54 ± 0.11	0.04 ± 0.005	+	
0.973	250	7	+		+		+		+		+		+	
		14	+		+		+		+		+		+	
		21	0.86 ± 0.25	0.05 ± 0.002	+		+		+		0.17 ± 0.009	0.01 ± 0.008	+	

Results are mean five replicates ± standard deviation and given in µg mycotoxin per milliliter (µg/mL) and mg mycotoxin related to g dry mass (mg/g); + detected with concentration lower than limit of quantitation.

Table S6. Penitrems production after 7, 14 and 21 days by *P. crustosum* in modified Czapek-Dox media supplemented with different inorganic nitrogen sources.

N source	Days	Pen A		Pen B		Pen C		Pen D		Pen E		Pen F	
		µg/mL	mg/g	µg/mL	mg/g	µg/mL	mg/g	µg/mL	mg/g	µg/mL	mg/g	µg/mL	mg/g
None added	7, 14, 21	ND		ND		ND		ND		ND		ND	
$NaNO_3$	7	12.19 ± 0.81	0.98 ± 0.069	1.43 ± 0.43	0.12 ± 0.015	+		2.04 ± 0.19	0.16 ± 0.036	2.22 ± 0.72	0.18 ± 0.021	+	
	14	15.21 ± 1.61	1.21 ± 0.043	1.56 ± 0.61	0.12 ± 0.12	+		3.01 ± 0.12	0.24 ± 0.071	2.76 ± 0.32	0.22 ± 0.012	+	
	21	19.21 ± 1.12	1.50 ± 0.023	2.94 ± 0.48	0.25 ± 0.004	+		3.97 ± 0.43	0.31 ± 0.009	4.86 ± 0.81	0.38 ± 0.008	+	
NH_4NO_3	7	14.78 ± 0.23	1.12 ± 0.082	1.75 ± 0.13	0.13 ± 0.017	ND		2.13 ± 0.24	0.16 ± 0.001	2.46 ± 0.34	0.18 ± 0.004	ND	
	14	19.19 ± 1.14	1.42 ± 0.081	3.62 ± 0.14	0.27 ± 0.009	+		3.21 ± 0.32	0.24 ± 0.040	3.16 ± 1.72	0.23 ± 0.054	+	
	21	22.32 ± 1.29	1.60 ± 0.055	4.46 ± 0.72	0.32 ± 0.004	+		4.32 ± 0.81	0.31 ± 0.085	4.46 ± 0.76	0.32 ± 0.011	+	
$(NH_4)_2SO_4$	7	9.12 ± 0.91	0.81 ± 0.073	1.02+0.11	0.09 ± 0.005	ND		2.14 ± 0.97	0.19 ± 0.005	1.68 ± 0.46	0.15 ± 0.001	ND	
	14	16.92 ± 1.12	1.47 ± 0.12	3.21 ± 0.21	0.28 ± 0.084	+		2.96 ± 0.28	0.26 ± 0.081	2.77 ± 0.43	0.24 ± 0.001	+	
	21	19.23 ± 0.68	1.62 ± 0.036	3.51 ± 0.12	0.31 ± 0.047	+		2.59 ± 0.17	0.22 ± 0.002	3.19 ± 0.58	0.27 ± 0.092	+	
NH_4Cl + $NaNO_3$	7	10.61 ± 0.14	0.79 ± 0.001	1.34 ± 0.22	0.10 ± 0.082	+		1.51 ± 0.54	0.11 ± 0.12	1.63 ± 0.71	0.12 ± 0.054	+	
	14	14.86 ± 1.38	1.1 ± 0.13	2.18 ± 0.97	0.16 ± 0.075	+		1.87 ± 0.36	0.14 ± 0.006	2.19 ± 0.91	0.16 ± 0.021	+	
	21	20.39 ± 2.54	1.5 ± 0.075	1.63 ± 0.31	0.12 ± 0.001	+		3.13 ± 0.84	0.23 ± 0.009	3.67 ± 0.12	0.27 ± 0.058	+	
NH_4Cl	7	15.11 ± 0.29	1.05 ± 0.25	1.75 ± 0.27	0.12 ± 0.009	+		2.56 ± 0.13	0.18 ± 0.009	3.12 ± 0.42	0.22 ± 0.001	+	
	14	22.78 ± 0.25	1.57 ± 0.032	1.47 ± 0.11	0.11 ± 0.001	+		3.99 ± 0.12	0.28 ± 0.002	4.12 ± 0.52	0.28 ± 0.13	+	
	21	26.19 ± 2.75	1.8 ± 0.026	2.51 ± 0.12	0.17 ± 0.001	+		4.98 ± 0.72	0.34 ± 0.006	5.15 ± 0.71	0.36 ± 0.049	+	

Results are mean five replicates ± standard deviation and given in µg mycotoxin per milliliter (µg/mL) and mg mycotoxin related to g dry mass (mg/g); ND: not detected; + detected with concentration lower than limit of quantitation.

Table S7. Penitrems production after 7, 14 and 21 days by *P. crustosum* in modified Czapek-Dox media supplemented with different organic nitrogen sources.

N source	Days	Pen A		Pen B		Pen C		Pen D		Pen E		Pen F	
		µg/mL	mg/g	µg/mL	mg/g	µg/mL	mg/g	µg/mL	mg/g	µg/mL	mg/g	µg/mL	mg/g
None added	7,14,21	ND		ND		ND		ND		ND		ND	
Urea	7	11.23 ± 0.71	0.67 ± 0.001	1.31 ± 0.38	0.08 ± 0.002	ND		3.01 ± 0.27	0.18 ± 0.001	2.42 ± 0.76	0.14 ± 0.001	ND	
	14	17.81 ± 0.32	1.04 ± 0.036	2.98 ± 0.73	0.18 ± 0.091	ND		3.12 ± 0.31	0.18 ± 0.054	3.24 ± 0.12	0.19 ± 0.093	ND	
	21	20.32 ± 1.28	1.12 ± 0.061	3.08 ± 0.53	0.17 ± 0.005	+		3.08 ± 0.74	0.17 ± 0.008	4.34 ± 0.83	0.24 ± 0.001	+	
Gly	7	11.52 ± 0.91	1.12 ± 0.006	1.35 ± 0.32	0.14 ± 0.006	+		1.23 ± 0.12	0.12 ± 0.086	1.86 ± 0.57	0.19 ± 0.004	+	
	14	17.44 ± 0.28	1.53 ± 0.63	3.01 ± 0.87	0.26 ± 0.009	+		3.21 ± 0.23	0.28 ± 0.001	3.17 ± 1.21	0.28 ± 0.023	+	
	21	23.43 ± 1.79	2.04 ± 0.073	3.44 ± 0.64	0.30 ± 0.006	+		4.02 ± 1.29	0.35 ± 0.004	3.67 ± 0.87	0.32 ± 0.002	+	
Ser	7	19.78 ± 2.12	1.72 ± 0.32	2.45 ± 0.12	0.21 ± 0.009	+		2.98 ± 0.87	0.26 ± 0.009	3.66 ± 0.21	0.32 ± 0.005	+	
	14	20.03 ± 1.95	1.71 ± 0.056	4.21 ± 1.23	0.36 ± 0.054	+		4.12 ± 0.45	0.35 ± 0.005	4.16 ± 0.82	0.35 ± 0.009	+	
	21	24.32 ± 1.42	2.04 ± 0.089	5.04 ± 0.51	0.42 ± 0.009	+		5.52 ± 1.27	0.46 ± 0.069	6.12 ± 0.53	0.51 ± 0.001	+	
Pro	7	12.52 ± 0.54	1.14 ± 0.013	1.23 ± 0.73	0.11 ± 0.023	+		2.34 ± 0.65	0.21 ± 0.002	2.51 ± 0.34	0.23 ± 0.043	+	
	14	19.11 ± 1.87	1.74 ± 0.009	3.83 ± 0.62	0.35 ± 0.009	+		4.82 ± 0.34	0.45 ± 0.001	4.24 ± 0.62	0.39 ± 0.001	+	
	21	24.34 ± 2.26	2.11 ± 0.062	4.12 ± 1.41	0.36 ± 0.019	+		4.23 ± 0.89	0.37 ± 0.023	4.86 ± 0.83	0.42 ± 0.008	+	
Arg	7	18.92 ± 0.63	1.53 ± 0.008	2.24 ± 0.12	0.18 ± 0.047	+		3.09 ± 1.22	0.25 ± 0.043	3.15 ± 0.21	0.26 ± 0.007	+	
	14	19.78 ± 0.92	1.61 ± 0.001	4.45 ± 0.11	0.36 ± 0.063	+		4.91 ± 0.23	0.40 ± 0.023	4.52 ± 0.38	0.37 ± 0.001	+	
	21	24.32 ± 0.75	1.94 ± 0.089	3.88 ± 0.12	0.31 ± 0.029	+		4.25 ± 0.12	0.34 ± 0.044	4.38 ± 0.33	0.35 ± 0.016	+	
Glu	7	18.36 ± 0.34	1.65 ± 0.52	2.15 ± 0.78	0.19 ± 0.014	+		3.52 ± 1.12	0.32 ± 0.031	3.78 ± 0.12	0.34 ± 0.028	+	
	14	29.17 ± 0.84	3.52 ± 0.12	8.32 ± 2.01	0.99 ± 0.087	+		7.01 ± 0.78	0.84 ± 0.013	7.29 ± 1.11	0.88 ± 0.009	+	
	21	38.12 ± 1.92	4.03 ± 0.284	7.52 ± 0.21	0.79 ± 0.031	1.71 ± 0.46	0.18 ± 0.053	8.11 ± 0.96	0.85 ± 0.022	8.67 ± 0.37	0.91 ± 0.003	1.42 ± 0.31	0.15 ± 0.017
Trp	7	9.69 ± 0.42	0.82 ± 0.008	1.51 ± 0.21	0.1 ± 0.009	+		1.87 ± 0.67	0.2 ± 0.009	1.86 ± 0.17	0.2 ± 0.01	+	
	14	23.28 ± 1.25	0.95 ± 0.014	3.5 ± 0.41	0.14 ± 0.002	+		2.91 ± 0.47	0.12 ± 0.006	3.82 ± 0.87	0.15 ± 0.01	+	
	21	27.67 ± 1.23	0.92 ± 0.052	4.54 ± 0.36	0.28 ± 0.020	+		4.89 ± 0.73	0.32 ± 0.046	5.12 ± 1.11	0.38 ± 0.001	+	

Results are mean five replicates ± standard deviation and given in µg mycotoxin per milliliter (µg/mL) and mg mycotoxin related to g dry mass (mg/g); ND: not detected; + detected with concentration lower than limit of quantitation.

Table S8. Penitrems production after 7, 14 and 21 days by *P. crustosum* in modified Czapek-Dox media supplemented with tryptophan.

Concentration of *Trp*, mM	Days	Pen A		Pen B		Pen C		Pen D		Pen E		Pen F	
		µg/mL	mg/g	µg/mL	mg/g	µg/mL	mg/g	µg/mL	mg/g	µg/mL	mg/g	µg/mL	mg/g
5	7	8.91 ± 0.13	0.8 ± 0.011	1.11 ± 0.18	0.1 ± 0.008	+		1.11 ± 0.94	0.1 ± 0.008	2.22 ± 0.18	0.2 ± 0.071	+	
	14	10.53 ± 0.25	0.9 ± 0.085	1.17 ± 1.12	0.1 ± 0.007	+		1.17 ± 0.13	0.1 ± 0.001	2.34 ± 0.21	0.2 ± 0.091	+	
	21	15.87 ± 1.42	1.4 ± 0.23	2.26 ± 0.54	0.2 ± 0.009	+		3.39 ± 0.78	0.3 ± 0.003	3.40 ± 0.85	0.3 ± 0.013	+	
10	7	9.69 ± 0.42	0.82 ± 0.008	1.51 ± 0.21	0.1 ± 0.009	+		1.87 ± 0.67	0.2 ± 0.009	1.86 ± 0.17	0.2 ± 0.01	+	
	14	23.28 ± 1.25	0.95 ± 0.014	3.5 ± 0.41	0.14 ± 0.002	+		2.91 ± 0.47	0.12 ± 0.006	3.82 ± 0.87	0.15 ± 0.01	+	
	21	27.67 ± 1.23	0.92 ± 0.052	4.54 ± 0.36	0.28 ± 0.020	+		4.89 ± 0.73	0.32 ± 0.046	5.12 ± 1.11	0.38 ± 0.001	+	
20	7	8.51 ± 0.52	0.8 ± 0.016	1.06 ± 0.18	0.1 ± 0.008	+		+		2.12 ± 0.13	0.2 ± 0.006	+	
	14	10.79 ± 0.26	0.9 ± 0.005	3.59 ± 0.79	0.3 ± 0.005	+		3.59 ± 0.56	0.3 ± 0.014	2.39 ± 1.36	0.2 ± 0.003	+	
	21	10.98 ± 0.87	0.8 ± 0.009	2.75 ± 0.32	0.2 ± 0.008	+		2.75 ± 0.42	0.2 ± 0.001	2.75 ± 0.98	0.2 ± 0.001	+	

Results are mean five replicates ± standard deviation and given in µg mycotoxin per milliliter (µg/mL) and mg mycotoxin related to g dry mass (mg/g); + detected with concentration lower than limit of quantitation.

Table S9. Penitrems production after 7, 14 and 21 days by *P. crustosum* in modified Czapek-Dox media supplemented with glycerol.

a_w	Concentration of Glycerol, g/L	Days	Pen A		Pen B		Pen C		Pen D		Pen E		Pen F	
			µg/mL	mg/g	µg/mL	mg/g	µg/mL	mg/g	µg/mL	mg/g	µg/mL	mg/g	µg/mL	mg/g
		7	19.62 ± 0.52	0.83 ± 0.005	2.83 ± 0.001	0.12 ± 0.009	ND		4.96 ± 1.07	0.21 ± 0.018	5.91 ± 1.12	0.25 ± 0.005	ND	
0.995	5	14	20.45 ± 0.14	0.87 ± 0.001	2.35 ± 0.104	0.10 ± 0.021	+		5.41 ± 1.39	0.23 ± 0.013	5.17 ± 0.13	0.22 ± 0.007	+	
		21	36.46 ± 0.76	1.55 ± 0.038	3.06 ± 0.35	0.13 ± 0.001	+		5.41 ± 1.56	0.23 ± 0.001	4.94 ± 0.1	0.21 ± 0.004	+	
		7	20.12 ± 0.38	0.83 ± 0.002	2.91 ± 0.17	0.12 ± 0.082	ND		4.13 ± 0.057	0.17 ± 0.009	5.33 ± 0.41	0.22 ± 0.001	ND	
0.993	10	14	20.37 ± 0.14	0.81 ± 0.003	2.77 ± 0.35	0.11 ± 0.001	+		5.28 ± 0.50	0.21 ± 0.001	5.28 ± 0.13	0.21 ± 0.001	+	
		21	37.87 ± 1.84	1.51 ± 0.02	3.08 ± 0.22	0.13 ± 0.002	+		5.22 ± 1.61	0.22 ± 0.01	4.99 ± 0.25	0.21 ± 0.001	+	
		7	44.71 ± 0.88	1.89 ± 0.014	4.49 ± 0.12	0.19 ± 0.055	+		7.33 ± 0.77	0.31 ± 0.029	7.33 ± 0.72	0.31 ± 0.034	ND	
0.992	15	14	45.30 ± 0.43	1.90 ± 0.019	4.53 ± 0.47	0.19 ± 0.003	+		7.62 ± 0.071	0.32 ± 0.002	7.15 ± 0.27	0.30 ± 0.021	+	
		21	45.54 ± 0.50	1.92 ± 0.421	4.51 ± 1.21	0.19 ± 0.025	+		7.82 ± 1.25	0.33 ± 0.018	6.88 ± 1.12	0.29 ± 0.011	+	
		7	11.78 ± 0.25	0.50 ± 0.026	4.71 ± 1.12	0.20 ± 0.001	+		4.94 ± 0.72	0.21 ± 0.01	5.42 ± 0.18	0.23 ± 0.003	ND	
0.990	30	14	20.03 ± 0.66	0.85 ± 0.001	3.29 ± 0.12	0.14 ± 0.002	+		6.13 ± 1.76	0.26 ± 0.002	6.36 ± 0.028	0.27 ± 0.001	+	
		21	25.03 ± 2.75	1.07 ± 0.009	4.21 ± 0.31	0.18 ± 0.008	+		6.08 ± 1.65	0.26 ± 0.009	6.78 ± 0.14	0.29 ± 0.002	+	
		7	5.69 ± 0.63	0.19 ± 0.001	2.72 ± 0.38	0.091 ± 0.001	+		4.49 ± 0.57	0.15 ± 0.007	3.59 ± 0.52	0.12 ± 0.004	+	
0.987	50	14	24.70 ± 6.01	0.79 ± 0.001	4.06 ± 1.82	0.13 ± 0.002	+		6.25 ± 0.61	0.2 ± 0.001	6.56 ± 0.17	0.21 ± 0.001	+	
		21	25.79 ± 0.62	0.82 ± 0.003	3.77 ± 1.22	0.12 ± 0.002	+		6.29 ± 0.52	0.2 ± 0.001	5.98 ± 0.89	0.19 ± 0.005	+	
		7	5.53 ± 0.66	0.18 ± 0.022	2.46 ± 0.46	0.08 ± 0.001	ND		2.76 ± 0.056	0.09 ± 0.031	1.84 ± 0.19	0.06 ± 0.002	ND	
0.986	70	14	10.44 ± 0.14	0.34 ± 0.081	1.53 ± 1.24	0.05 ± 0.032	+		1.53 ± 0.58	0.05 ± 0.011	1.22 ± 0.44	0.04 ± 0.003	+	
		21	13.95 ± 0.80	0.45 ± 0.034	1.55 ± 0.28	0.05 ± 0.071	+		0.93 ± 0.78	0.03 ± 0.002	2.17 ± 0.16	0.07 ± 0.003	+	
		7	3.65 ± 0.33	0.07 ± 0.003	+		ND		0.52 ± 0.006	0.01 ± 0.071	1.04 ± 0.13	0.02 ± 0.0001	ND	
0.985	100	14	3.85 ± 0.41	0.08 ± 0.003	ND		ND		ND		0.48 ± 0.13	0.01 ± 0.0001	ND	
		21	9.03 ± 0.87	0.18 ± 0.001	ND		ND		+		+		ND	
		7	1.97 ± 0.76	0.03 ± 0.007	ND		ND		ND		ND		ND	
0.985	150	14	2.63 ± 0.22	0.05 ± 0.001	ND		ND		ND		ND		ND	
		21	7.61 ± 0.52	0.13 ± 0.002	ND		ND		ND		ND		ND	
		7	1.17 ± 0.13	0.02 ± 0.009	ND		ND		ND		ND		ND	
0.971	200	14	1.31 ± 0.62	0.02 ± 0.036	ND		ND		ND		ND		ND	
		21	6.19 ± 0.38	0.09 ± 0.001	ND		ND		ND		ND		ND	
		7	+		ND		ND		+		ND		ND	
0.958	250	14	+		ND		ND		ND		ND		ND	
		21	5.61 ± 0.14	0.08 ± 0.001	ND		ND		ND		ND		ND	
		7	ND		ND		ND		ND		ND		ND	
0.949	300	14	ND		ND		ND		ND		ND		ND	
		21	ND		ND		ND		ND		ND		ND	

Results are mean five replicates ± standard deviation and given in µg mycotoxin per milliliter (µg/mL) and mg mycotoxin related to g dry mass (mg/g); ND: not detected; + detected with concentration lower than limit of quantitation.

Table S10. Penitrems production after 7, 14 and 21 days by *P. crustosum* in modified Czapek-Dox media supplemented with NaCl.

a_w	Concentration of NaCl, g/L	Days	Pen A		Pen B		Pen C		Pen D		Pen E		Pen F	
			µg/mL	mg/g	µg/mL	mg/g	µg/mL	mg/g	µg/mL	mg/g	µg/mL	mg/g	µg/mL	mg/g
0.993	0	7	ND	ND	ND		ND		ND		ND	ND	ND	
		14	6.08 ± 0.21	0.54 ± 0.01	ND		ND		ND		1.01 ± 0.97	0.09 ± 0.001	ND	
		21	8.31 ± 0.31	0.72 ± 0.006	+		ND		+		1.51 ± 0.12	0.12 ± 0.003	ND	
0.993	5	7	7.77 ± 0.25	0.78 ± 0.003	+		ND		+		1.29 ± 0.57	0.13 ± 0.002	ND	
		14	14.36 ± 0.14	1.45 ± 0.042	+		ND		+		2.27 ± 0.17	0.23 ± 0.005	ND	
		21	13.11 ± 1.77	2.02 ± 0.651	+		ND		+		2.73 ± 0.67	0.42 ± 0.081	ND	
0.991	10	7	9.61 ± 0.88	0.97 ± 0.004	+		ND		+		1.48 ± 0.49	0.15 ± 0.006	ND	
		14	17.02 ± 0.99	1.73 ± 0.072	+		ND		+		3.24 ± 0.49	0.33 ± 0.044	ND	
		21	13.77 ± 4.55	1.96 ± 0.046	+		ND		+		2.95 ± 0.51	0.42 ± 0.062	ND	
0.990	20	7	5.44 ± 0.14	0.48 ± 0.003	+		ND		+		0.91 ± 0.28	0.08 ± 0.001	ND	
		14	7.19 ± 0.52	0.63 ± 0.001	+		ND		+		1.02 ± 0.028	0.09 ± 0.002	ND	
		21	7.02 ± 0.51	0.53 ± 0.009	+		ND		+		1.19 ± 0.022	0.09 ± 0.004	ND	
0.987	30	7	5.69 ± 0.29	0.51 ± 0.005	+		ND		+		0.78 ± 0.14	0.07 ± 0.002	ND	
		14	5.77 ± 0.57	0.47 ± 0.003	+		ND		+		0.85 ± 0.11	0.07 ± 0.001	ND	
		21	5.25 ± 0.25	0.42 ± 0.004	+		ND		+		0.87 ± 0.17	0.07 ± 0.008	ND	
0.983	40	7	+		ND		ND		ND		+		ND	
		14	3.86 ± 1.47	0.27 ± 0.003	ND		ND		ND		0.57 ± 0.28	0.04 ± 0.001	ND	
		21	4.52 ± 1.75	0.31 ± 0.007	+		ND		+		0.58 ± 0.31	0.04 ± 0.001	ND	
0.981	50	7	+		ND		ND		ND		+		ND	
		14	+		ND		ND		ND		+		ND	
		21	0.77 ± 0.53	0.04 ± 0.001	ND		ND		ND		+		ND	
0.973	60	7	+		ND		ND		ND		+		ND	
		14	+		ND		ND		ND		+		ND	
		21	+		ND		ND		ND		+		ND	
0.959	70	7	ND		ND		ND		ND		ND		ND	
		14	ND		ND		ND		ND		ND		ND	
		21	+		ND		ND		ND		+		ND	
0.955	80	7	ND		ND		ND		ND		ND		ND	
		14	ND		ND		ND		ND		ND		ND	
		21	+		ND		ND		ND		+		ND	
0.953	100	7	ND		ND		ND		ND		ND		ND	
		14	ND		ND		ND		ND		ND		ND	
		21	ND		ND		ND		ND		ND		ND	

Results are mean five replicates ± standard deviation and given in µg mycotoxin per milliliter (µg/mL) and mg mycotoxin related to g dry mass (mg/g); ND: not detected; + detected with concentration lower than limit of quantitation.

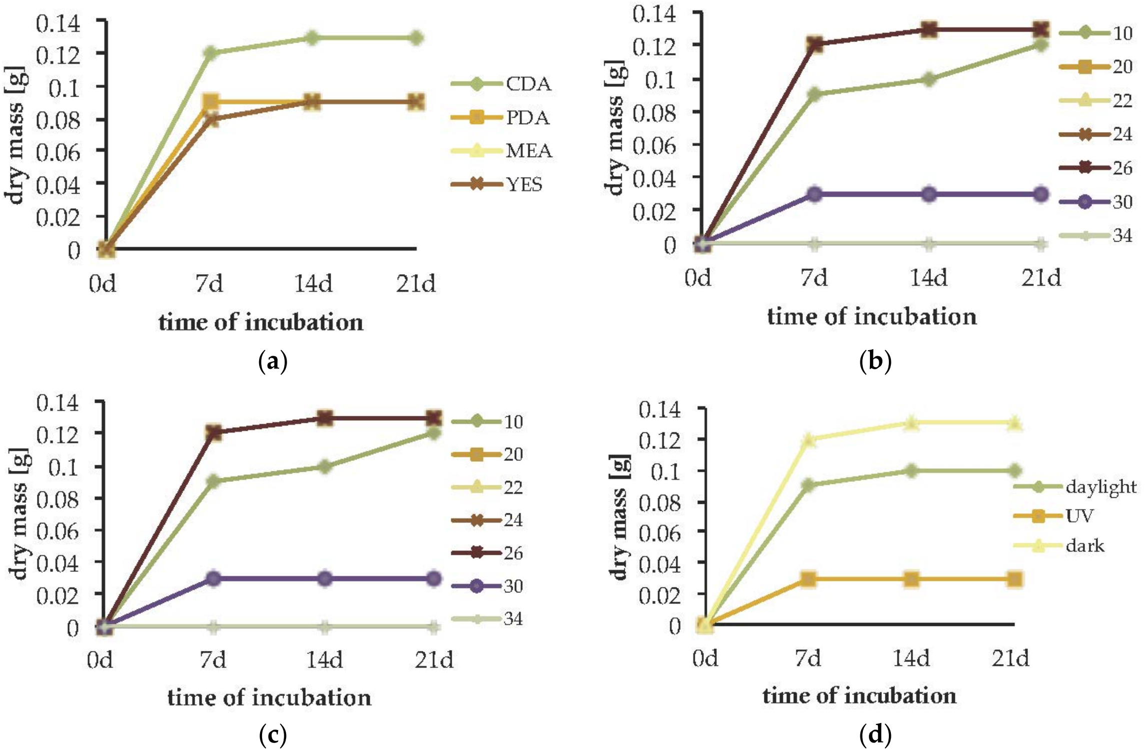

Figure S1. Effect of media (**a**), temperature (**b**), pH (**c**) and light (**d**) on *P. crustosum* growth after 7, 14 and 21 days of incubation.

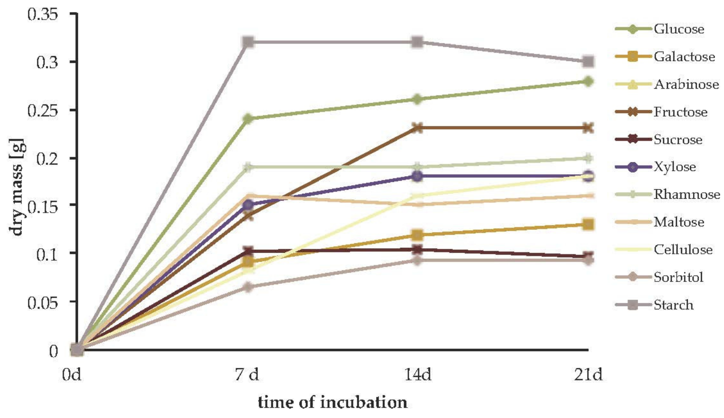

Figure S2. The growth of *P. crustosum* cultured on modified CDA supplemented with different C sources after 7, 14 and 21 days of incubation.

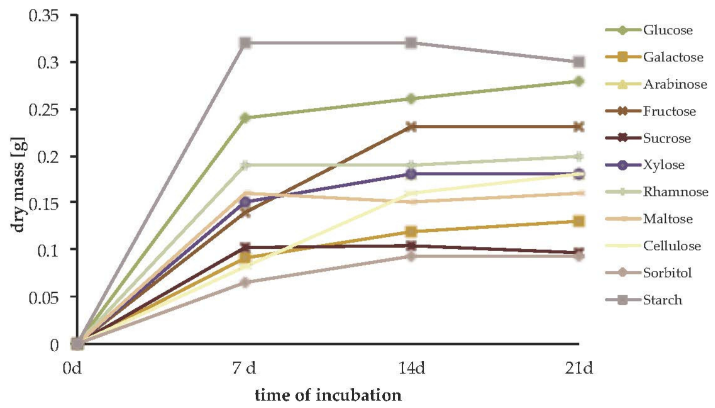

Figure S3. The growth of *P. crustosum* cultured on modified CDA supplemented with different concentration of glucose after 7, 14 and 21 days of incubation.

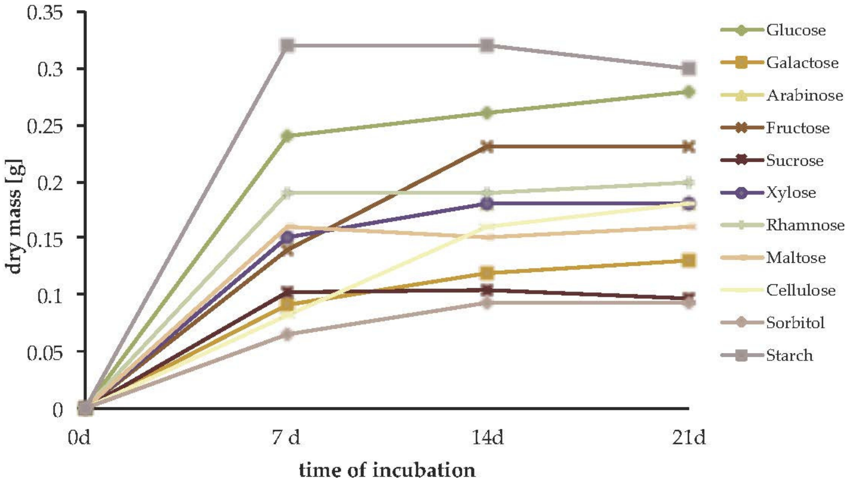

Figure S4. The growth of *P. crustosum* cultured on modified CDA supplemented with different inorganic nitrogen sources after 7, 14 and 21 days of incubation.

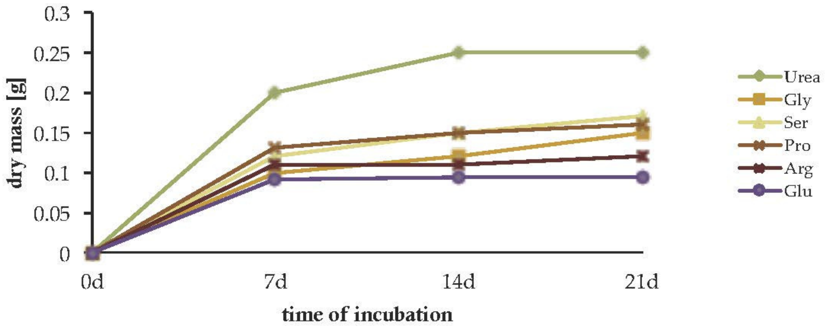

Figure S5. The growth of *P. crustosum* cultured on modified CDA supplemented with different organic nitrogen sources after 7, 14 and 21 days of incubation.

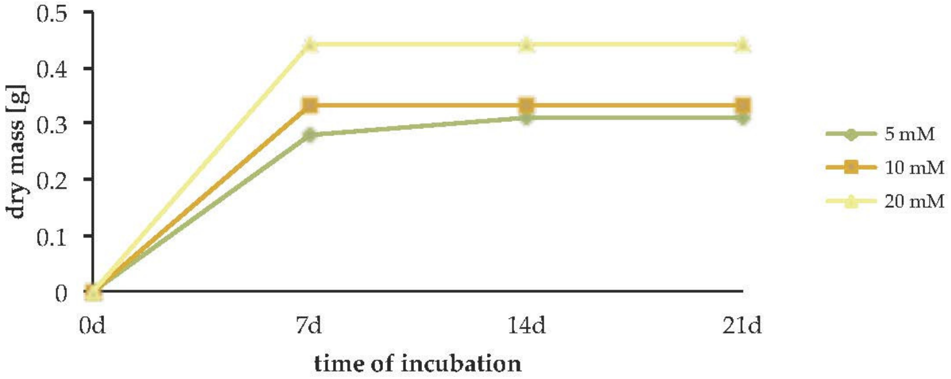

Figure S6. The growth of *P. crustosum* cultured on modified CDA supplemented with different concentration of tryptophan after 7, 14 and 21 days of incubation.

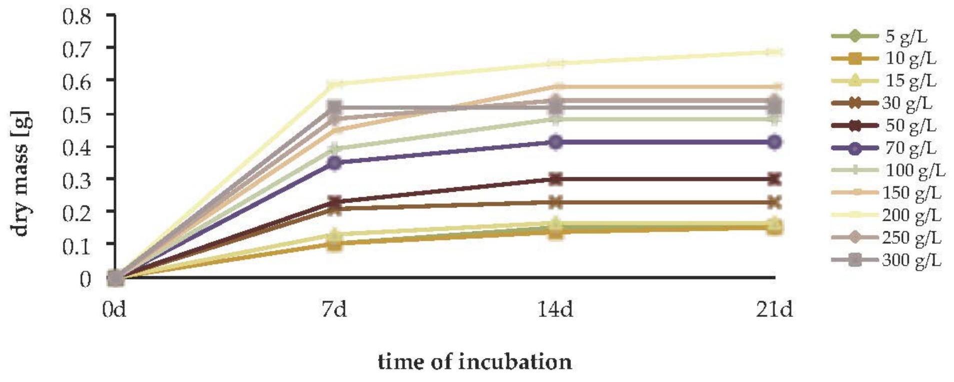

Figure S7. The growth of *P. crustosum* cultured on modified CDA supplemented with different concentration of glycerol after 7, 14 and 21 days of incubation.

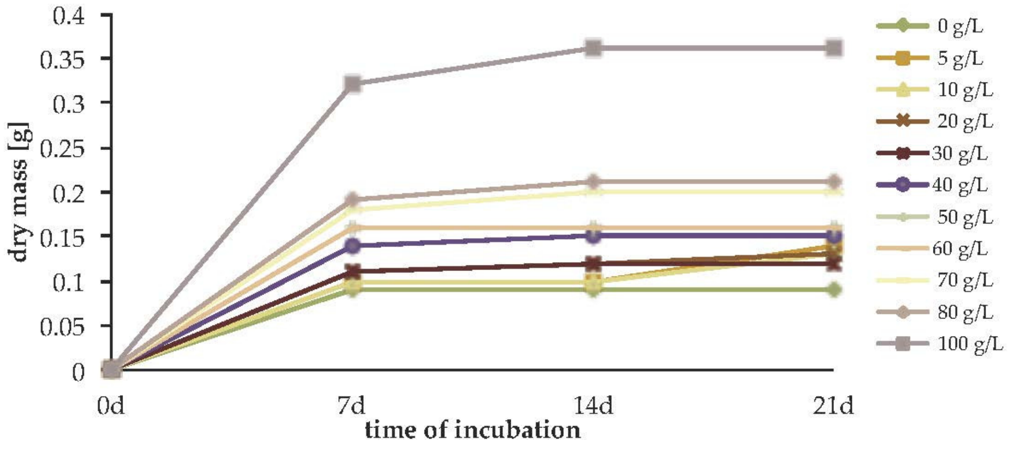

Figure S8. The growth of *P. crustosum* cultured on modified CDA supplemented with different concentration of NaCl after 7, 14 and 21 days of incubation.

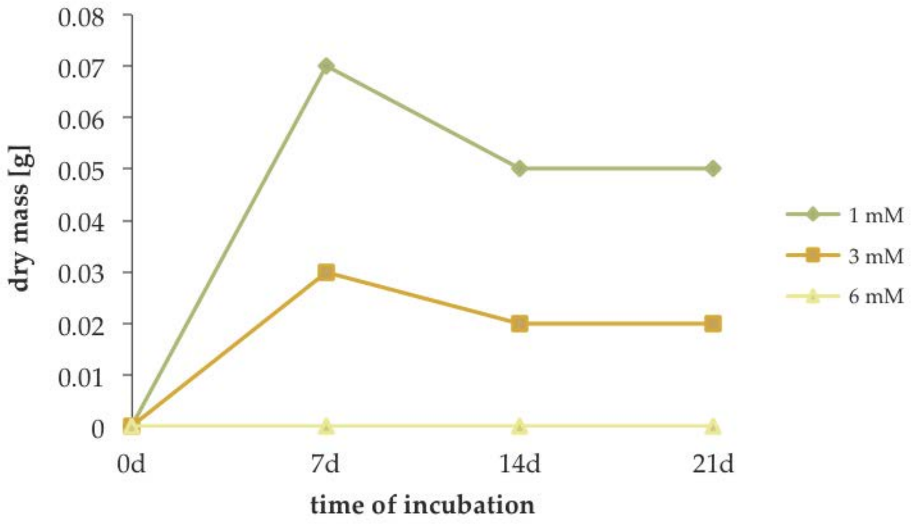

Figure S9. The growth of *P. crustosum* cultured on modified CDA supplemented with different concentration of H_2O_2 after 7, 14 and 21 days of incubation.
